# Quantitative, multiplexed, targeted proteomics for ascertaining variant specific SARS-CoV-2 antibody response

**DOI:** 10.1016/j.crmeth.2022.100279

**Published:** 2022-08-12

**Authors:** Ivan Doykov, Tomas Baldwin, Justyna Spiewak, Kimberly C. Gilmour, Joseph M. Gibbons, Corinna Pade, Catherine J. Reynolds, Áine McKnight, Mahdad Noursadeghi, Mala K. Maini, Charlotte Manisty, Thomas Treibel, Gabriella Captur, Marianna Fontana, Rosemary J. Boyton, Daniel M. Altmann, Tim Brooks, Amanda Semper, UK COVIDsortium Investigators, Hakam Abbass, Aderonke Abiodun, Mashael Alfarih, Zoe Alldis, Daniel M. Altmann, Oliver E. Amin, Mervyn Andiapen, Jessica Artico, João B. Augusto, Georgina L. Baca, Sasha N.L. Bailey, Anish N. Bhuva, Alex Boulter, Ruth Bowles, Rosemary J. Boyton, Olivia V. Bracken, Ben O’Brien, Tim Brooks, Natalie Bullock, David K. Butler, Gabriella Captur, Olivia Carr, Nicola Champion, Carmen Chan, Aneesh Chandran, Tom Coleman, Jorge Couto de Sousa, Xose Couto-Parada, Eleanor Cross, Teresa Cutino-Moguel, Silvia D’Arcangelo, Rhodri H. Davies, Brooke Douglas, Cecilia Di Genova, Keenan Dieobi-Anene, Mariana O. Diniz, Anaya Ellis, Karen Feehan, Malcolm Finlay, Marianna Fontana, Nasim Forooghi, Sasha Francis, Joseph M. Gibbons, David Gillespie, Derek Gilroy, Matt Hamblin, Gabrielle Harker, Georgia Hemingway, Jacqueline Hewson, Wendy Heywood, Lauren M. Hickling, Bethany Hicks, Aroon D. Hingorani, Lee Howes, Ivie Itua, Victor Jardim, Wing-Yiu Jason Lee, Melaniepetra Jensen, Jessica Jones, Meleri Jones, George Joy, Vikas Kapil, Caoimhe Kelly, Hibba Kurdi, Jonathan Lambourne, Kai-Min Lin, Siyi Liu, Aaron Lloyd, Sarah Louth, Mala K. Maini, Vineela Mandadapu, Charlotte Manisty, Áine McKnight, Katia Menacho, Celina Mfuko, Kevin Mills, Sebastian Millward, Oliver Mitchelmore, Christopher Moon, James Moon, Diana Muñoz Sandoval, Sam M. Murray, Mahdad Noursadeghi, Ashley Otter, Corinna Pade, Susana Palma, Ruth Parker, Kush Patel, Mihaela Pawarova, Steffen E. Petersen, Brian Piniera, Franziska P. Pieper, Lisa Rannigan, Alicja Rapala, Catherine J. Reynolds, Amy Richards, Matthew Robathan, Joshua Rosenheim, Cathy Rowe, Matthew Royds, Jane Sackville West, Genine Sambile, Nathalie M. Schmidt, Hannah Selman, Amanda Semper, Andreas Seraphim, Mihaela Simion, Angelique Smit, Michelle Sugimoto, Leo Swadling, Stephen Taylor, Nigel Temperton, Stephen Thomas, George D. Thornton, Thomas A. Treibel, Art Tucker, Ann Varghese, Jessry Veerapen, Mohit Vijayakumar, Tim Warner, Sophie Welch, Hannah White, Theresa Wodehouse, Lucinda Wynne, Dan Zahedi, James C. Moon, Kevin Mills, Wendy E. Heywood

**Affiliations:** 1 Translational Mass Spectrometry Research Group, Genetics & Genomic Medicine Department, UCL Institute of Child Health, London, UK; 2 Great Ormond Street Biomedical Research Centre, UCL Institute of Child Health London; 3 Great Ormond Street Children’s Hospital NHS Foundation Trust, Great Ormond Street, London WC1N 3JH, UK; 4 Blizard Institute, Barts and the London School of Medicine and Dentistry, Queen Mary University of London, London, UK; 5 Department of Infectious Disease, Imperial College London, London, UK; 6 Division of Infection and Immunity, University College London, London, UK; 7 St. Bartholomew’s Hospital, Barts Health NHS Trust, London, UK; 8 Institute of Cardiovascular Science, University College London, London, UK; 9 Royal Free London NHS Foundation Trust, Pond Street, London NW3 2QG, UK; 10 Lung Division, Royal Brompton and Harefield Hospitals, Guy’s and St Thomas’ NHS Foundation Trust, London, UK; 11 Department of Immunology and Inflammation, Imperial College London, London, UK; 12 UK Health Security Agency, Porton Down, UK

**Keywords:** complement: immunoglobulin, SARS-CoV-2, vaccination, proteomics, COVID-19, variant of concern, mass spectrometry, omicron variant, delta variant

## Abstract

Determining the protection an individual has to severe acute respiratory syndrome coronavirus-2 (SARS-CoV-2) variants of concern (VoCs) is crucial for future immune surveillance, vaccine development, and understanding of the changing immune response. We devised an informative assay to current ELISA-based serology using multiplexed, baited, targeted proteomics for direct detection of multiple proteins in the SARS-CoV-2 anti-spike antibody immunocomplex. Serum from individuals collected after infection or first- and second-dose vaccination demonstrates this approach and shows concordance with existing serology and neutralization. Our assays show altered responses of both immunoglobulins and complement to the Alpha (B.1.1.7), Beta (B.1.351), and Delta (B.1.617.1) VoCs and a reduced response to Omicron (B1.1.1529). We were able to identify individuals who had prior infection, and observed that C1q is closely associated with IgG1 (r > 0.82) and may better reflect neutralization to VoCs. Analyzing additional immunoproteins beyond immunoglobulin (Ig) G, provides important information about our understanding of the response to infection and vaccination.

## Highlights

• Multiplexed targeted proteomics is a viable alternative to ELISA-based testing
• The immunocomplex to SARS-CoV-2 spike is altered against variants of concern (VoCs)
• IgA is an indicator of prior SARS-CoV-2 infection
• C1q can reflect neutralization against VoCs better than IgG1

## Motivation

Assays for measuring serum antibody responses are typically limited to measurement of a total or single immunoglobulin isotype. The antibody response is far more complex, with multiple immunoglobulin classes, isotypes, and complement factors involved. This is a potential wealth of information that is typically understudied and missed by existing tests. The global COVID-19 pandemic has highlighted the need to understand better the immune response in respect to vaccine development and emerging new severe acute respiratory syndrome coronavirus-2 (SARS-CoV-2) variants. Using the ability of tandem mass spectrometry to multiplex and directly and accurately measure the antibody complex, we devised an alternative assay to capture this valuable information.

## Graphical abstract

**Figure:**
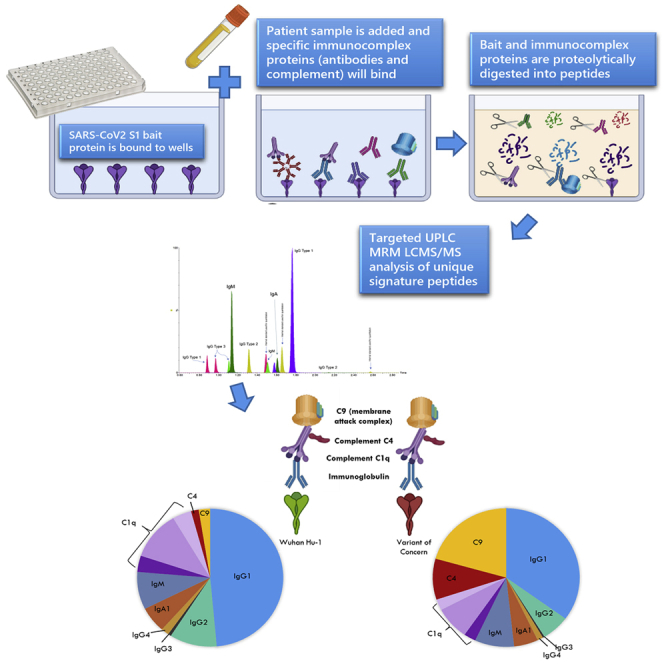

## Teaser

Doykov et al. present an assay that reveals changes in immunoglobulin classes, subtypes, and complement in response to vaccination, infection, and variants of concern. The assay can provide insight into an individual’s immune response and aid vaccine design and delivery. It can also be applied to other antigens and diseases.

## Introduction

After the first cases of severe acute respiratory syndrome coronavirus-2 (SARS-CoV-2) were identified in late 2019, 2020–2021 saw the development and rollout of the world’s fastest and largest global vaccination programs. However, with potential waning immunity over time (Gaebler et al., 2021) and the impact of infection from emerging variants of concern (VoCs) (Reynolds et al., 2021a), it is apparent that there is a need for better and more informative testing (Abbasi, 2021). This will help determine the clinical need for booster vaccination and timing of the boost itself. First-generation tests were rolled out at scale but are largely based on simple non-specific binding to the prototypic Wuhan Hu-1 spike sequence. It is now clear that these methods overestimate the actual protective immunity against VoCs (Reynolds et al., 2021a, 2021b, 2022). Current technologies that directly measure accepted correlates of protection such as neutralizing antibodies (nAbs) scale poorly for clinical utility, while serological approaches (ELISA or electrochemiluminescent immunoassay [ECLIA]) measure only part of the antibody response and omit measurement of effector Fc antibody functions such as complement involvement (Ju et al., 2020; Nie et al., 2020; Yu et al., 2020).

To aid in understanding the antibody response to SARS-CoV-2, we have developed a methodology that includes a “bait and capture” system, followed by a multiplexed and targeted proteomic liquid chromatography-tandem mass spectrometry (LC-MS/MS) analyses. The combination of immunocapture with the multiplexing capability to look at multiple proteins involved in the immune response and high accuracy of mass spectrometry quantitation makes this an extremely powerful and more informative combination. In addition, tandem mass spectrometers are also routinely used for small molecule clinical assays in most UK pathology laboratories and are therefore platforms that could be utilized for targeted proteomic assays. It is only recently, with improving technology, that they are becoming recognized for their potential clinical application for multiplex protein analysis (Smit et al., 2021).

In this work, we describe how we have used this assay to compare with previously determined immune correlates (Reynolds et al., 2021a, 2021b, 2022) in serial samples, in response to vaccination, infection, and an individual’s potential protection against VoCs. This analysis was performed using serum samples from the COVIDsortium study (Manisty et al., 2021a, 2021b; Reynolds et al., 2020, 2021b, 2022; Treibel et al., 2020), where previously detailed longitudinal immunological analysis had been carried out. This unique cohort included healthcare workers (HCWs) with and without laboratory-confirmed SARS-CoV-2 infection, during the first UK wave with the Wuhan Hu-1 strain and after one- and two-dose vaccination (Pfizer/BioNTech BNT162b2) (Manisty et al., 2021a; Reynolds et al., 2020, 2021a, 2021b). Our analyses demonstrate that the conventional measurement of immunoglobulin (Ig)G1 is insufficient to determine an individual’s complete immuno-response or protection due to infection and vaccination. We show that responses to infection and vaccination can be heterogeneous from person to person, and, by broadening the portfolio of those biomarkers involved in the monitoring of the immune response, this assay provides a more informative picture of immune responses. This assay could be used to estimate an individual’s immune potency against VoCs but also aid in design of future vaccine trials.

## Results

### The development of a multiplex LC-MS/MS assay for measuring antibody mediated response to SARS-CoV-2 spike antigen

**Figure 1:**
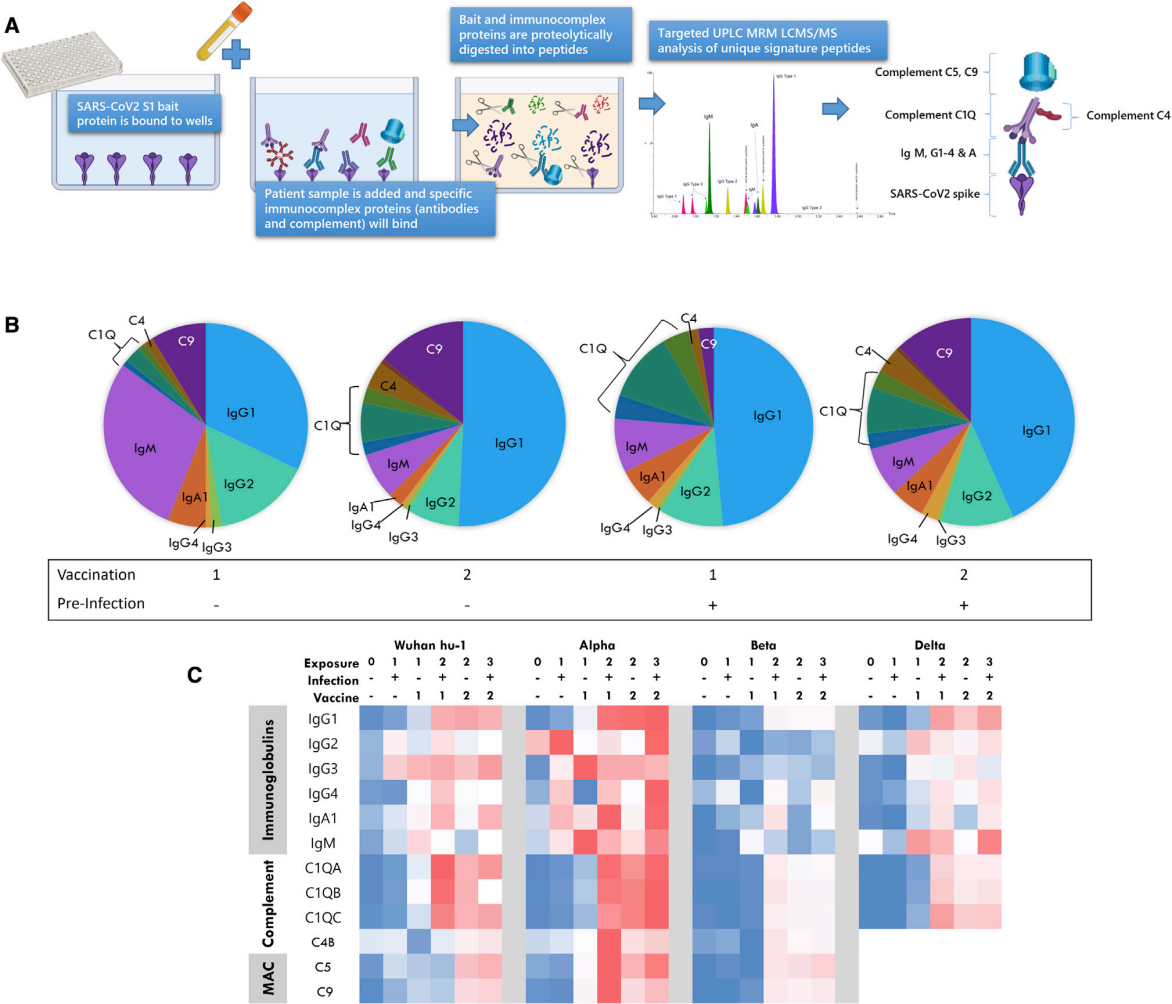
Principle of the targeted LC-MS/MS immunocomplex assay (A) A summarized workflow and schematical representation of the bait-capture LC-MS/MS assay. (B) Composition of the immunocomplex at different vaccination and pre-infection stages. Mean values used for each protein. (C) Heatmap of all proteins measured in the multiplex as determined by normalized mean values of the protein ratioed to spike peptide against each S1 bait variant and vaccination group. MAC, membrane attack complex. Blue to red color scale indicates lowest to highest values.

The multiplex assay developed is significantly more sophisticated and informative because of its ability to quantitate, simultaneously and accurately, all major antibody species and their subclasses, as well as key components of the downstream complement pathway. The rationale behind inclusion of complement proteins is based on their involvement in formation of antigen-antibody complexes. Figure 1A is a schematic representation of how the assay captures and analyses a patient serum immunocomplex specific to the SARS-CoV-2 spike region. A recombinant SARS-CoV-2 S1 spike protein from any VoC is first bound to a 96-well plate in a simple procedure described below. Patient serum is incubated with the bait for 60 min to capture the immunocomplex, and non-specific proteins are washed away. All immunocaptured proteins, including the spike, are trypsin digested and the unique signature peptides analyzed by a targeted LC-MS/MS analyses. The assay was capable of identifying and quantitating the immunoglobulins IgG1, 2, 3, and 4; IgA1; IgM; and the complement factors C1q, C4b, and C9 using 10 μL of serum with a CV range of 1.7%–13.6% for high response quality controls (QCs) and 1%–15.3% for low-response QCs. To improve reproducibility over existing immunodetection assays, results were expressed as a ratio of each immunological protein to the SARS-CoV-2 spike (using a S1 peptide common to all variants). This improves the CV percentage by a factor of ∼10-fold; i.e., a standalone value CV for IgG1 is reduced from 13.8% to 3.4% if the value is ratioed to spike bait. Internal monitoring of bait binding also provides a quality assurance for plate preparation.

Figure 1B shows how the profile of the immunocomplex changes according to vaccination and prior infection. A heatmap summary of the proteins included in the immunocomplex and their response to exposure and variants is given in Figure 1C, which summarizes the increase of the immunocomplex with increasing exposure against all SARS-CoV-2 variants. However, a notable reduction of detectable immune activity against the Beta VoC can be observed compared with other variants analyzed. A strength of our assay is to measure all major antibody types simultaneously, allowing comparison of inter-individual and exposure isotype heterogeneity. When all components of the immunocomplex are viewed collectively (Figure S1), a wide variation in those with two or more antigen exposures can be observed with clear outliers. Closer inspection of the outliers highlights that these individuals have an atypical immunocomplex profile, with one interesting individual who has a dominant IgG4 response. IgG4 is associated with anti-inflammatory properties as it can undergo fab-arm exchange, thereby limiting effector functions (van der Neut Kolfschoten et al., 2007). This indicates that a one-size-fits-all strategy using the current Elecsys assays does not provide us with enough information if we are to determine and study an individual’s current immune status.

As proof of principle, we also demonstrated that this methodology can also be applied to the much less invasive measurement of antibodies and immune response proteins (compared with collecting blood serum) using dried blood spots, saliva, or saliva adsorbed onto Guthrie card blood collection strips or “lollipops” (Figure S2).

### Comparison with the gold standard S1 receptor binding domain (RBD) serology assay and authentic live virus neutralization

We compared our assay with the gold standard serology and authentic live virus neutralization assays at three timepoints, sampling responses 8 weeks after natural infection with Wuhan Hu-1 during the first UK wave and 3 weeks after the first- and second-dose vaccination. This enabled us to evaluate and compare our assay after one to three antigen exposures using the same existing peer-reviewed, published underpinning datasets analyzed in these cohorts (Manisty et al., 2021a; Reynolds et al., 2020, 2021a, 2021b). We compared results from our assay with those obtained by second-generation serology, which measures a total response to spike bait and not a specific antibody (anti-SARS-CoV-2 spike ECLIA assay [Elecsys, Roche Diagnostics], performed by the UK Health Security Agency [UKHSA], Porton Down, UK).

**Figure 2:**
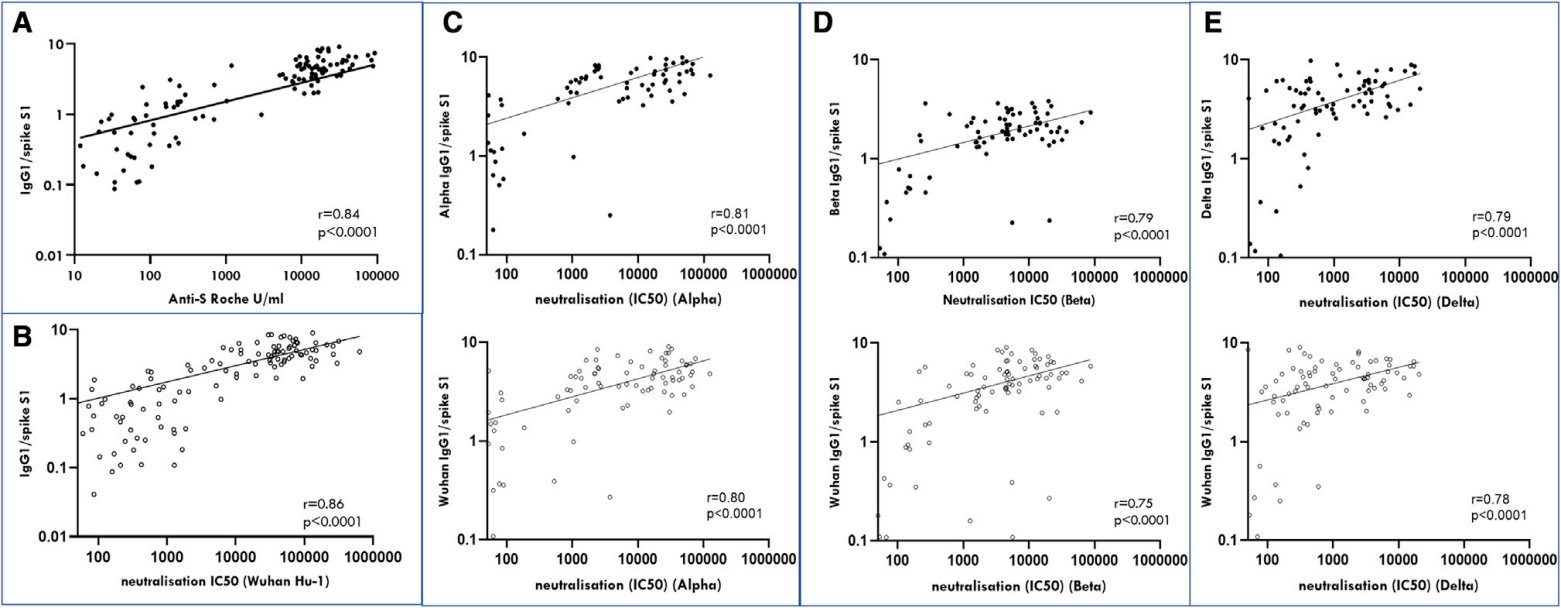
Comparison of LC-MS/MS IgG1 levels with other serology method and live viral neutralization (A) Roche Elecsys Anti-S assay versus IgG1 LC-MS/MS. (B) Wuhan Hu-1 neutralizing antibodies versus Wuhan S1 IgG1 LC-MS/MS. (C) Alpha neutralizing antibodies versus Alpha and Wuhan S1 IgG1 LC-MS/MS. (D) Beta neutralizing antibodies versus Beta and Wuhan S1 IgG1 LC-MS/MS. (E) Delta neutralizing antibodies versus Delta and Wuhan S1 IgG1 LC-MS/MS. Significance determined by Spearman correlation. n = 141 in total for n = 24 infection-naive group, n = 23 previous infection group at pre, first, and second vaccination time points.

In agreement with other studies (Dogan et al., 2021; Patil et al., 2021), our test also confirmed in most individuals that IgG1 is the main responsive immunoglobulin. Figure 2A shows that IgG1 correlates well (r = 0.84) across a broad range (10–100,000 U/mL) with the Elecsys Anti-S assay and requires no dilution and repeat analyses for high-titer individuals.

The ability to measure all the components of the immunocomplex demonstrated that IgG1 showed the strongest correlation with live-virus neutralization data (nAbs) (Figure S3) followed by C1q. Figures 2B–2E show that comparison of IgG1 against each S1 variant bait correlates well with corresponding nAbs (r = 0.79–0.86). Comparison using only IgG1 Wuhan Hu-1 S1 against neutralization data for each variant shows only slightly less correlation against Alpha, Beta, and Delta VoCs (r = 0.75–0.80). This confirms our high-throughput assay demonstrated excellent correlation with conventional neutralizing cell-based assays as well and on par with the commercial Roche Anti-S assay.

### Determining the immuno-response and protection to both Wuhan Hu-1 and other VoCs

**Figure 3:**
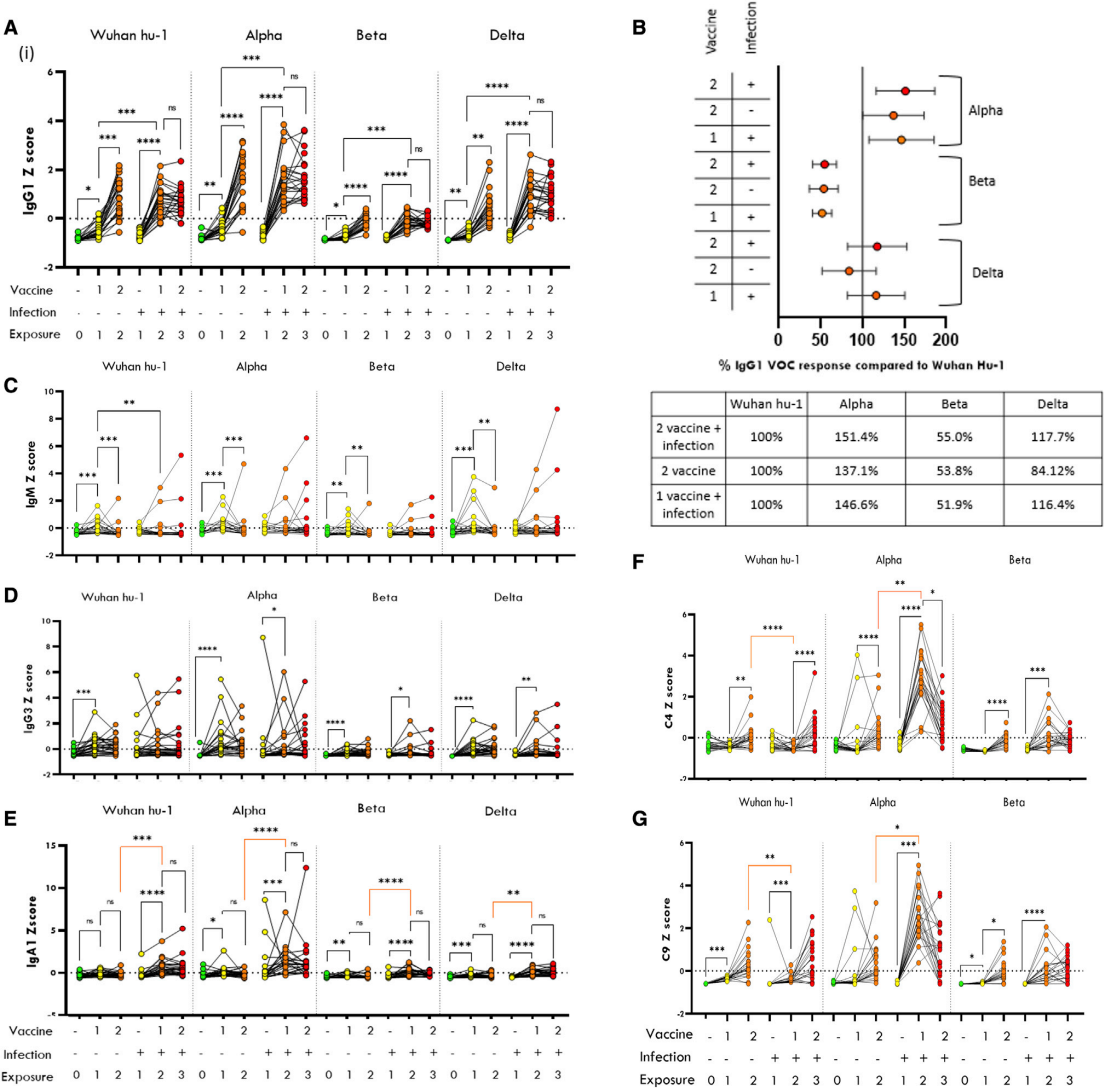
Application of immunocomplex assay to vaccinated participant cohort (A) The comparison of IgG1 levels binding to each VoC spike according to vaccine status in healthcare workers, who have had (+) or not had (−) a prior SARS CoV-2 infection. Results are shown for the wild-type Wuhan Hu-1, Alpha, Beta, and Delta spike variants. (B) Summaries of average percentage of the IgG1 response to the VoC compared with Wuhan Hu-1. Comparison of other proteins of immunocomplex according to vaccine status for (C) IgM, (D) IgG3, (E) IgA1, (F) complement C4, and (G) complement C9. Vaccination groups colored according to exposure status. Green, no exposure; yellow, first exposure (X1); orange, second exposure (X2), and red, third exposure(X3). Data normalized for VoC comparison. Significance determined by non-parametric ANOVA and Spearman correlation p values ∗ ≤0.05, ∗∗ ≤ 0.01, ∗∗∗ ≤ 0.001, ∗∗∗∗ ≤ 0.0001. Colored significance bars indicate change due to pre-infection. n = 24 infection-naive group, n = 23 previous infection group.

Our results for IgG1 (Figure 3A) using the LC-MS/MS assay are in line with our published findings that two exposures, either via a two-dose vaccination protocol or natural infection and one-dose vaccination, have the same effect whereby the second antigen exposure increases anti-Wuhan spike antibody levels on average 3- to 10-fold (Manisty et al., 2021b; Reynolds et al., 2021a, 2021b). In concordance with the gold-standard S1-RBD binding and authentic live virus neutralization assays (Reynolds et al., 2021b; Reynolds et al., 2021b), we found no increase in anti-Wuhan Hu-1 S1 IgG1 responses from the second vaccine dose in previously infected HCWs. This suggests an antibody ceiling is achieved at third antigen exposure.

To determine an individual’s antibody reactivity against existing and new VoCs, the S1 protein from each VoC (Alpha, Beta, Delta, Omicron) was bound in three separate wells to compare patients’ serum antibody binding capabilities and immune responses. IgG1 values were then presented as a percentage of binding capability to the original Wuhan Hu-1 strain and vaccine target, to give the patients a measure of IgG1 reactivity against each VoC (Figure 3B). We observe more than a 37%–51% greater response against the Alpha S1 compared with the Wuhan Hu-1, as described previously, which is slightly enhanced by previous natural infection (Reynolds et al., 2021a). IgG1 response to Delta S1 is similar to Wuhan Hu-1 (±17%) and is also enhanced by previous infection (Figure 3B). IgG1 response to Beta S1 only elicits a 52%–55% response relative to the protection against the Wuhan Hu-1 VoC, which is likely due to combined effects of the K417N and N501Y mutations in the Beta spike (Zhou et al., 2021).

Changes in IgM (Figure 3C) are only observed in the infection-naive group for all variants where the levels increase at first vaccination, but levels are lower at second vaccination, confirming IgM only changes in response to first exposure. No significant group changes related to exposure or infection were observed for IgG2 or IgG4 in most individuals. However, some individuals had notable and significantly higher levels of IgG2 and IgG4 (Figure S1), which indicates that a more personalized medicine approach may be applicable for some individuals. IgG3 is always observed, albeit at lower levels and as would be expected (Ferrante et al., 1990). IgG3 appears to increase at first exposure in the vaccine-naive group and, unlike IgM, the levels appear to stay elevated with further exposure. The pre-infection group show a small significant increase at first vaccination only for the VoC (Figure 3D). Of note are differences in the levels of IgA1 (Figure 3E). No change is seen in the infection-naive group against the Wuhan Hu-1 bait, but an increase is observed for the pre-infection group at first vaccination. However, for the VoC baits after first vaccination, we observed a small but significant increase of IgA1 in the infection-naive group, although this response in the group who had a previous infection was more marked. When comparing with double-exposure groups (two-dose vaccination infection naive versus one-dose vaccination with prior infection), we see clearly a greater IgA1 response (p < 0.01) in those individuals who had a natural infection (Figure 3E).

### Understanding antibody protection beyond immunoglobulins: Determining and quantitating the complement response against Wuhan Hu-1 and VoCs

Current tests only determine IgG or total response. Using our assay, we are able to also determine the significant and important contribution from the complement system. Complement C4 levels demonstrated an average 2- to 6-fold significant increase against the native Wuhan Hu-1. This was observed only at the second vaccination stage in both groups, indicating a response to vaccine but, interestingly, not from exposure to SARS-CoV-2 in natural infection (Figure 3F). Complement C9 also appears to significantly increase with vaccination first dose against Wuhan-Hu1 by an average 3.8- to 5.2-fold (Figure 3G). However, the C4 and C9 response to the Alpha and Beta VoCs, respectively, were observed to be markedly different than that to the native Wuhan-Hu1. The response to the Alpha shows that both complement C4 and C9 increase greatly in the pre-infection group after first vaccination (9.6-fold and 93-fold, respectively). However, this response is not as great after a second vaccination, with a lower 3.8-fold and 46-fold increase, for C4 and C9 respectively compared with the pre-vaccine group. A similar but lower-level response pattern is observed for Beta. This indicates pre-infection elicits greater C4 and C9 against VoC binding after a second antigen exposure.

**Figure 4:**
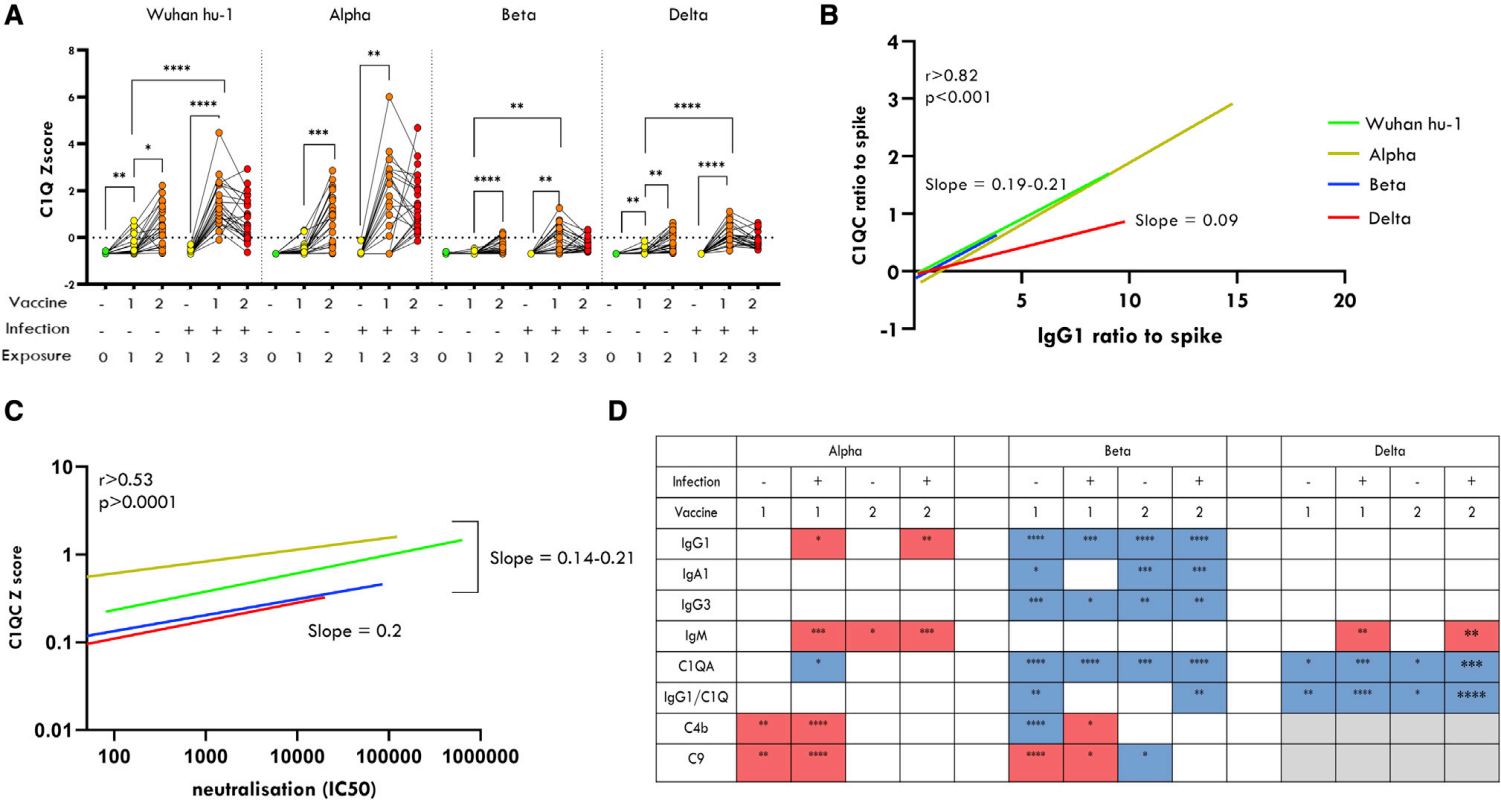
C1q response to variant S1 protein (A) The comparison of C1Q levels binding to each VoC spike according to vaccine status in healthcare workers, who have had (+) (n = 23) or not had (−) (n = 24) a prior SARS CoV-2 infection. Results are shown for the wild-type Wuhan Hu-1, Alpha, Beta, and Delta spike variants. Linear regression based on correlation showing changes in the slope/ratio for Delta for (B) C1Q versus IgG1, and no change in slope/ratio for Delta for (C) C1Q versus nAbs (log scale) analysis performed on all four vaccination groups combined (n = 94). (D) A summary of significant changes of the immunocomplex due to VoC compared with Wuhan Hu-1 response. Blue decreased and red increased. Vaccination groups colored according to exposure status. Green, no exposure; yellow, first exposure; orange, second exposure; and red, third exposure. Significance determined by non-parametric ANOVA and Spearman correlation p values ∗ ≤ 0.05, ∗∗ ≤ 0.01, ∗∗∗ ≤ 0.001, ∗∗∗∗ ≤ 0.0001.

Complement C1q binding to all variants increases after exposure similar to that of IgG1 (Figure 4A) but the levels observed are lower against the Beta and Delta. While it is not significant, we did observe less C1q binding at third exposure, where binding increased 42-fold after first vaccination, but only a 31-fold increase was observed at second vaccination in the pre-infected group. The similar pattern of C1q binding to IgG1 is likely due to the direct interaction of C1q with the Fc region of IgG; when compared, we observe a significant correlation (r > 0.8) between IgG1 and C1q for all variants. However, when comparing correlation between variants, a reduction in the ratio of IgG1 to C1q against the Delta VoC, and, to a lesser extent against the Beta VoC, were observed (Figure 4B). This suggests potentially a reduced interaction of C1q with IgG1 against Beta and Delta VoCs.

Considering the relationship of IgG1 to nAbs (r > 0.79; Figure 2), we also looked at the C1q relationship to nAb levels for each variant (Figure 4C). Unlike with the IgG1 levels, we did not observe any significant differences in the gradient/ratio of C1q to nAbs compared with the other variants. This indicates a better association of C1q to nAbs than IgG1 for the Delta VoC.

To demonstrate this further, when we combine the nAb data for all variants and then compare with IgG1, and also C1q, we can observe that C1q has an overall stronger correlation with nAb than IgG1 (r = 0.62 versus 0.71) (Figures S4A–S4B). If we look at the data as a ratio of C1q to IgG1 across the vaccine groups, we see this ratio is increased with previous exposure to infection (p < 0.01 all variants), but this significantly declines at third exposure (p < 0.001 all variants) (Figure S4C). When comparing across the variants, we see that the Beta and Delta groups show a significant reduced ratio compared with Wuhan Hu-1, with the exception of the second-exposure groups, where the ratios are normal against the Beta VoC (Figure S4C). The significance of this observation is unknown and requires further investigation but could relate to a change in the antibody function or maturation that is affected by prior infection and variant type.

The VoC response compared with Wuhan-hu1 is summarized in Figure 3H, showing an overall reduced response to the Beta VoC, although with a greater C9 response at first vaccination. A greater IgM response against Alpha and Delta is detected in the pre-infected groups and is likely due to a first exposure to a variant. These changes likely relate to the contribution of non-neutralizing antibodies that use complement-mediated lysis, which can be driven by IgM (*17*).

### Application of the multiplex assay to the Omicron variant with triple-vaccinated samples

**Figure 5:**
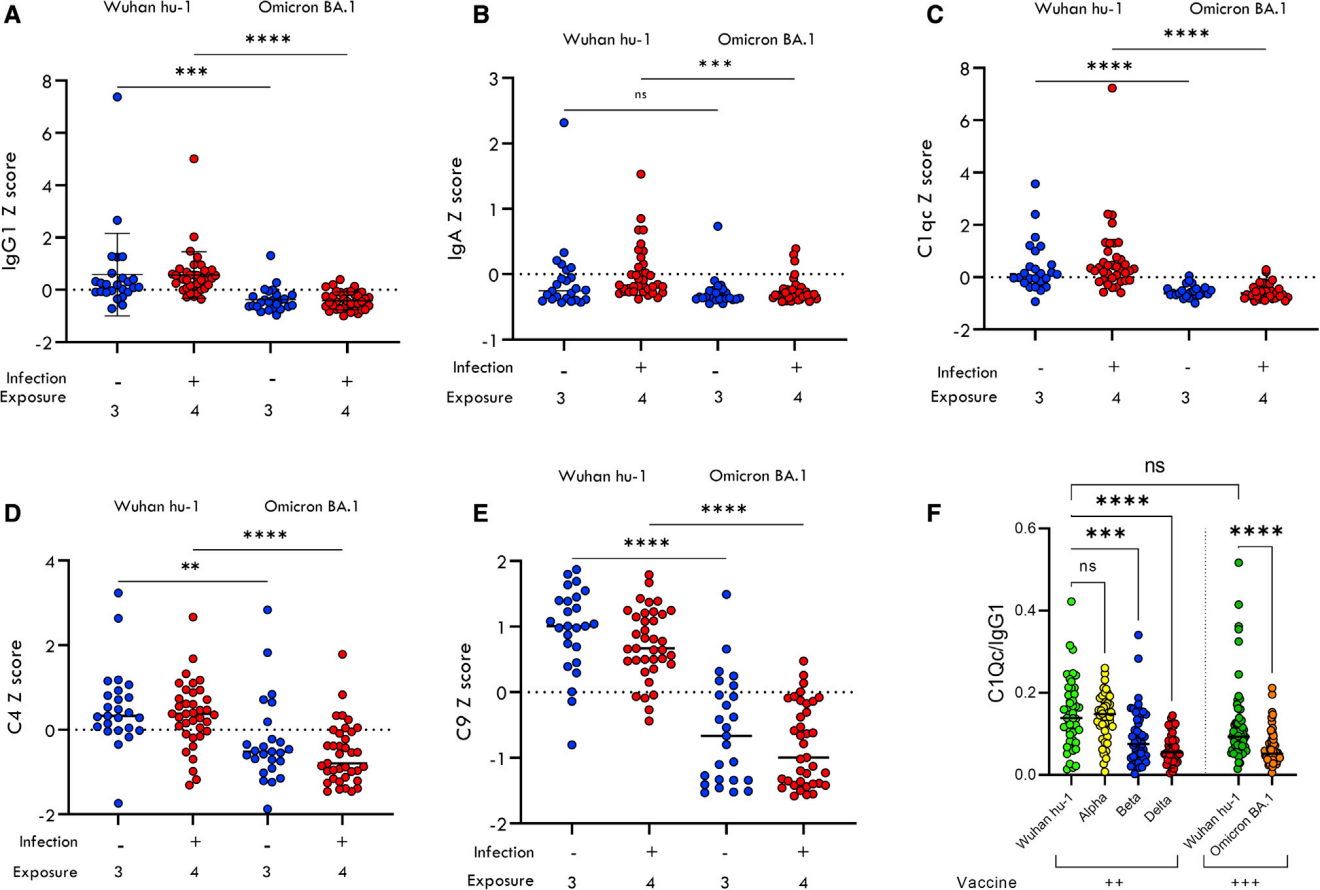
Response of triple-vaccinated HCWs to the Omicron BA.1 VoC Comparison of triple vaccinated with or without prior infection against S1 protein from Wuhan Hu-1 or Omicron BA.1 infection; naive n = 25 (blue), prior infected n = 36 (red). Mean ± 1 standard deviation are indicated on all plots. (A) IgG1. (B) IgA. (C) C1q. (D) C4. (E) C9. (F) Comparison of ratio of C1q/IgG1 between VoCs for double (n = 47) and triple (n = 76) vaccinated. Significance determined by non-parametric Kruskal-Wallis test.p-values ∗ ≤ 0.05, ∗∗ ≤ 0.01, ∗∗∗ ≤ 0.001, ∗∗∗∗ ≤ 0.0001

The Omicron variant B.1.1529, which emerged in late November 2021, is the most genetically divergent variant to occur (Saxena et al., 2022), and, at the time of writing, BA.2 is rapidly becoming the main SARS-CoV-2 variant infecting individuals world-wide. Using recombinant spike S1 protein, we modified the assay and applied the multiplex assay to a separate cohort of HCW serum samples from patients who had all received a third Pfizer/BioNTech BNT162b2 dose. This cohort of samples were from HCWs that were either infection naive or had been previously infected by ancestral Wuhan Hu-1, Alpha, or Delta variants. Figure 5A shows a reduction of IgG1 binding to Omicron S1 compared with the Wuhan Hu-1 (p < 0.001). IgA1, which was typically higher in pre-infected samples, also demonstrated a reduced binding to Omicron in the prior infected group (Figure 5B). No changes were observed for IgG2, IgG3, and IgM, although a small degree of reduced binding was observed for IgG4 for both groups (Figure S4E). The complement proteins C1q, C4, and C9 were all shown to bind with a greatly reduced capacity against Omicron S1 protein (Figures 5C–5E). When comparing the ratio of C1q to IgG1 with the other variants, we see a lower but non-significant trend with the Wuhan Hu-1 after the third dose, with a significantly reduced ratio for Omicron, similar to that of the Delta VoC (Figure 5F).

## Discussion

Standard SARS-CoV-2 serological assays are becoming less effective as a representation of a correlate of protection against variants (Reynolds et al., 2021a), and there is a need for a more informative test for determining this information. This is due to the lack of specificity of current tests, which may either measure a total response to bait antigen such as the Roche Elecsys assay or total IgG. A measure of total response of all proteins with affinity for the spike protein will include all other components of the immunocomplex, such as other classes of immunoglobulins and complement. However, these conventional assays do not tell us how much these immune proteins contribute individually and are extremely important if we are to understand more fully the mechanisms of protection against SARS-CoV-2 variants. While the total response has been adequate for monitoring response to the original Wuhan Hu-1, it is no longer adequate against VoCs and in populations with an evolving immune response to multiple exposures (Reynolds et al., 2021a).

Our multiplexed approach is capable of looking at all immune-reactive proteins individually and demonstrates that other classes of immune proteins, such as IgA and the complement system, also significantly contribute to an individual’s overall immune reactivity against VoCs, all of which would be missed by conventional testing.

Just looking specifically at IgG1, if we use the Wuhan Hu-1 response as a baseline, we see a slight significant increase of IgG1 against the Alpha VoC (Figures 2B and 4D), which could be due to a more open conformation of the RBD domain in the Alpha spike (Yang et al., 2021). However, we observe no change for the Delta VoC. In addition, and confirming previous findings, pre-infection has been shown to significantly boost antibody response of the first dose of the vaccination (Anichini et al., 2021; Ebinger et al., 2021; Reynolds et al., 2021b). However, our data indicate that a double vaccine dose in the infection naive still does not achieve mean values of those who had pre-infection. Thus the combination of infection plus vaccination may provide a potentially higher immunity versus that of a conventional two-dose vaccination protocol. This effect is more noticeable for Delta exposure (Figure 2B), and previous reports have shown that prior infection does reduce the rate of Delta breakthrough infections (Kim et al., 2021).

However, contrary to our observation of greater or unaltered IgG1, it has been shown that nAbs are lower against VoCs (Reynolds et al., 2021a). This indicates IgG1 is less of a reflection of nAb ability against the VoC. This is likely due to a greater contribution of non-neutralizing antibodies against the VoC that cannot be differentiated by IgG1 measurement.

The evaluation of bound antibodies and complement fixation beyond just IgG1 in our assay revealed a surprisingly large degree of heterogeneity in response to exposure, with some individuals having completely altered immunoglobulin profiles (Figure S1). The relevance of some patients having a lower IgG1 but higher IgG2 and IgG4 responses is unknown, but this observation would be overlooked using conventional testing. This could also have relevance in future studies of inter-individual and longitudinal responses to continued exposure of SARS-CoV-2 and vaccines. This ability to define an individual’s immunoglobulin profile and determine the variability of an individual’s immunological make up is both intriguing and potentially very important, particularly in different clinical scenarios such as disease severity and mortality (Della-Torre et al., 2021; Patil et al., 2021; Perez-Toledo et al., 2021).

Unlike other assays that are available, our platform also measures the immunocomplex binding from the less well-understood but very important complement system. Complement activation can contribute to anti-viral defense by the classical or lectin pathway leading to neutralization by viral opsonization and lysis (Jayasekera et al., 2007; Kunnakkadan et al., 2019; Schiela et al., 2018; Vasantha et al., 1988). Previous observations from another study, which also looked at other components of the immunocomplex against SARS-CoV-2 RBD, also highlighted the relevance of C4, C3, and terminal complement complex (TCC, or membrane attack complex) deposition in relation to disease severity (Jarlhelt et al., 2021). They confirmed that deposition of C4, C3, and TCC is mediated by antibodies binding to epitopes on RBD, as complement deposition was almost completely absent post depletion of RBD antibodies in convalescent plasma samples. This was thought to be driven by IgG as complement increased with IgG in response to disease severity. However, against the variants, our findings using S1 spike protein show complement C4 and C9 are greater against variants at initial exposure. This could be due to the contribution of non-neutralizing antibodies, as studies on parainfluenza virus show that complement-mediated neutralization is likely to be a mechanism of non-neutralizing antibody action driven by IgM (Vasantha et al., 1988). Our findings corroborate this, as we observed a greater IgM response against Alpha and Delta, which was also accompanied by greater C4 and C9 binding (Figure 4D). However, there is likely the presence of non-neutralizing IgG1 as well due to the fact that IgG1 levels are unchanged against the Alpha and Delta variants (Figure 2), but there are reduced neutralizing antibodies (Reynolds et al., 2021a).

One of the more interesting findings in the analyses of the immunocomplex was that of the role of C1q. C1q directly interacts with the Fc portion of immunoglobulins and is required for initiation of the complement cascade. In our analysis, C1q binding appears to behave independently from the other complement components (Figure 4), showing a reduction in binding against the Beta and Delta VoCs (Figure 4A) as opposed to the increase we observe for C4 and C9 binding. Interestingly, for the Delta VoC, C1q was reduced relative to IgG1 but not relative to nAbs. This indicates that C1q could be a better surrogate indicator of nAb protection against SARS-CoV-2 variants than IgG1. Previous work has shown that heat inactivation, which would inactivate complement, appears not to affect IgG reactivity to the RBD domain (Amanat et al., 2020) but may affect its neutralization capability (Pastorino et al., 2020). This confirms that, while complement is not essential, it may have a role in neutralization. Further weight to this observation comes from Mehlhop et al. (2009), who showed that C1q increases the potency of antibodies against West Nile virus by modulating the stoichiometric requirements for neutralization. Therefore, it is possible that complement could potentially contribute to protection against VoC by C1q augmenting neutralizing antibodies and non-neutralizing antibodies using the classical complement pathway for viral opsonization. If indeed C1q binding is more specific to nAbs, then the altered ratio between C1q and IgG1 for the Delta VoC, which we observed in this study, again could be explained by a greater proportion of non-neutralizing IgG1 antibodies that are present. This finding of the C1q association with nAbs merits further investigation to confirm whether the IgG1 to C1q relationship could be an indirect way of determining neutralizing and non-neutralizing antibody responses to VoCs.

The most recent B.1.1529 (Omicron) response was also evaluated in a separate triple-vaccinated cohort of HCWs and compared with the wild-type Wuhan Hu-1. Unlike with other VoCs, a significantly reduced response was observed for IgG1, as well as IgA1, IgG4, and complement binding. This is in accordance with previous work that confirmed neutralization is reduced against the Omicron VoC (Dejnirattisai et al., 2022), even after a booster vaccination (Yu et al., 2022). In addition, a lack of increased IgM and C4 and C9 complement response that we see for other variants was not apparent for Omicron. Considering that Omicron is the most genetically distinct VoC, with over 30 mutations in its spike protein (Wang and Cheng, 2022), it is likely there is also a lack of non-neutralizing antibodies that recognize the spike due to the greater number of mutations. This may explain the reduced C4 and C9 complement, which may be associated to non-neutralizing antibody-mediated viral opsonization by complement (Vasantha et al., 1988).

Our approach using multiplex LC-MS/MS could provide a valuable platform to better enable research in this area. While the LC-MS/MS multiplex assay is a research-standard assay, it was designed so it can be easily translated for use in a clinical laboratory setting (Smit et al., 2021). The information obtained will allow us to understand in greater detail an individual’s antibody protection or be used in vaccine design. Furthermore this “bait, capture, and mass spectrometry approach can also have applications beyond SAR-CoV-2 for other infectious diseases, or even immune response to novel treatments and autoimmunity.

### Limitations of the study

Our findings also uncover an area of antibody-mediated immunity that is little understood, and complement function is far more complex than what we can relay in our study. One of the limitations with our assay is that we are not able to determine functional complement activation, although precise quantitative detection may be able to funnel further investigation. Another limitation is the constant changing of the spike protein sequence in emerging variants. As we use a common spike peptide to standardize, there is the future possibility this will change with a new VoC. In this study, our data show only a snapshot of the antibody response approximately 3 weeks after vaccination in non-hospitalized healthcare workers. Further studies to characterize the effect over time, infection from different variants, infection severity, and how the “immunocomplex signature” changes with age would give us a greater understanding of the evolving antibody-mediated immune response to SARS-CoV-2.

## Consortia

The members of The UK COVIDsortium are Hakam Abbass, Aderonke Abiodun, Mashael Alfarih, Zoe Alldis, Daniel M. Altmann, Oliver E. Amin, Mervyn Andiapen, Jessica Artico, João B. Augusto, Georgina L. Baca, Sasha N.L. Bailey, Anish N. Bhuva, Alex Boulter, Ruth Bowles, Rosemary J. Boyton, Olivia V. Bracken, Ben O’Brien, Tim Brooks, Natalie Bullock, David K. Butler, Gabriella Captur, Olivia Carr, Nicola Champion, Carmen Chan, Aneesh Chandran, Tom Coleman, Jorge Couto de Sousa, Xose Couto-Parada, Eleanor Cross, Teresa Cutino-Moguel, Silvia D’Arcangelo, Rhodri H. Davies, Brooke Douglas, Cecilia Di Genova, Keenan Dieobi-Anene, Mariana O. Diniz, Anaya Ellis, Karen Feehan, Malcolm Finlay, Marianna Fontana, Nasim Forooghi, Sasha Francis, Joseph M. Gibbons, David Gillespie, Derek Gilroy, Matt Hamblin, Gabrielle Harker, Georgia Hemingway, Jacqueline Hewson, Wendy Heywood, Lauren M. Hickling, Bethany Hicks, Aroon D. Hingorani, Lee Howes, Ivie Itua, Victor Jardim, Wing-Yiu Jason Lee, Melaniepetra Jensen, Jessica Jones, Meleri Jones, George Joy, Vikas Kapil, Caoimhe Kelly, Hibba Kurdi, Jonathan Lambourne, Kai-Min Lin, Siyi Liu, Aaron Lloyd, Sarah Louth, Mala K. Maini, Vineela Mandadapu, Charlotte Manisty, Áine McKnight, Katia Menacho, Celina Mfuko, Kevin Mills, Sebastian Millward, Oliver Mitchelmore, Christopher Moon, James Moon, Diana Muñoz Sandoval, Sam M. Murray, Mahdad Noursadeghi, Ashley Otter, Corinna Pade, Susana Palma, Ruth Parker, Kush Patel, Mihaela Pawarova, Steffen E. Petersen, Brian Piniera, Franziska P. Pieper, Lisa Rannigan, Alicja Rapala, Catherine J. Reynolds, Amy Richards, Matthew Robathan, Joshua Rosenheim, Cathy Rowe, Matthew Royds, Jane Sackville West, Genine Sambile, Nathalie M. Schmidt, Hannah Selman, Amanda Semper, Andreas Seraphim, Mihaela Simion, Angelique Smit, Michelle Sugimoto, Leo Swadling, Stephen Taylor, Nigel Temperton, Stephen Thomas, George D. Thornton, Thomas A. Treibel, Art Tucker, Ann Varghese, Jessry Veerapen, Mohit Vijayakumar, Tim Warner, Sophie Welch, Hannah White, Theresa Wodehouse, Lucinda Wynne, and Dan Zahedi.

## STAR★Methods

### Key resources table

**Table:** 

REAGENT or RESOURCE	SOURCE	IDENTIFIER
**Biological samples**		

Human sera	COVIDsortium Healthcare Workers bioresource	NCT04318314

**Chemicals, peptides, and recombinant proteins**		

Wuhan Hu-1 SARS-CoV-2 spike protein (S1)	Genscript	Z03501
Alpha VOC B.1.1.7 SARS-CoV-2 (2019-nCoV) Spike S1 (HV69-70 deletion, N501Y, D614G)-His Recombinant Protein	Sino biological	40591-V08H7
Beta VOC B.1.1351 SARS-CoV-2 Spike protein (S1, E484K, K417N, N501Y, His Tag	Genscript	Z03531-1
Delta VOC B.1.617.2 (T19R, G142D, E156G, 157–158 deletion, L452R, T478K, D614G, P681R) Protein (His Tag)	Sino biological	40591-V08H19
Omicron (B.1.1.529/Omicron) Spike Glycoprotein (S1), Sheep Fc-Tag (HEK293) A67V, H69del, V70del, T95I, G142D, V143del, Y144del, Y145del, N211del, L212I, ins214EPE, G339D, S371L, S373P, S375F, K417N, N440K, G446S, S477N, T478K, E484A, Q493R, G496S, Q498R, N501Y, Y505H, T547K, D614G, H655Y	Native antigen	REC32006-100
IgG protein standard	Sigma Aldrich	I4506

**Critical commercial assays**		

Cobas® SARS-CoV-2 reverse transcriptase polymerase chain reaction (RT-PCR) test	ROCHE	09425217001A-01
Anti-SARS-CoV-2 ELISA	EUROIMMUN	EI 2606–9601 G
Elecsys® Anti-SARS-CoV-2	ROCHE	09 203 095 190

**Deposited data**		

Panorama database	https://panoramaweb.org	SARCOV2immunocomplex
Mendeley Data	https://doi.org/10.17632/csx49c78c6.2	This paperMendeley Data, v2

**Software and algorithms**		

Skyline open source software V.21.2	MacLean B, Tomazela DM, Shulman N, Chambers M, Finney GL, Frewen B, Kern R, Tabb DL, Liebler DC, MacCoss MJ. Skyline: an open source document editor for creating and analyzing targeted proteomics experiments. Bioinformatics. 2010 Apr 1;26(7):966–8. https://doi.org/10.1093/bioinformatics/btq054. Epub 2010 Feb 9. PMID: 20147306; PMCID: PMC2844992.	https://skyline.ms/project/home/software/Skyline/begin.view
Graphpad Prism v9	www.graphpad.com	www.graphpad.com/scientific-software/prism/

### Resource availability

#### Lead contact

Further information and requests for resources and reagents should be directed to and will be fulfilled by the lead contact, Ivan Doykov (i.doykov@ucl.ac.uk).

#### Materials availability

This study did not generate any new materials or reagents.

### Experimental model and subject details

#### Ethics statement

Human sera were obtained from the COVIDsortium Healthcare Workers bioresource (Manisty et al., 2021a; Reynolds et al., 2020, 2021b; Treibel et al., 2020) which is approved by the ethical committee of UK National Research Ethics Service (20/SC/0149) and registered on ClinicalTrials.gov (NCT04318314). The study conformed to the principles of the Helsinki Declaration, and all subjects gave written informed consent.

#### COVIDsortium healthcare worker participants

SARS-CoV-2 infection (by the Wuhan Hu-1 strain) of study participants was determined by baseline and weekly nasal RNA stabilizing swabs and Roche cobas® SARS-CoV-2 reverse transcriptase polymerase chain reaction (RT-PCR) test as well as baseline and weekly serology using the EUROIMMUN Anti-SARS-CoV2 enzyme-linked immunosorbent assay (ELISA) and ROCHE Elecsys® Anti-SARS-CoV-2 electrochemiluminescence immunoassay (ECLIA). Antibody ratios >1.1 were considered test positive for the EUROIMMUN SARS-CoV-2 ELISA and >1 was considered test positive for the ROCHE Elecsys anti-SARS-CoV-2 ECLIA following Public Health England evaluation (Public Health England, 2021; Manisty et al., 2021a, 2021b; Reynolds et al., 2021b; Treibel et al., 2020)

The previously reported^13,14^ cross-sectional, case-controlled vaccine sub-study (n = 51) collected samples at a mean/median timepoint of 22d and 20d after administration of the first and second dose of the mRNA vaccine, BNT162b2. This vaccine sub-study recruited HCW previously enrolled in the 16–18 week sub-study (Manisty et al., 2021b). This included 25 HCW (mean age 44 yr, 60% male) with previous laboratory defined evidence of WT SARS-CoV-2 infection and twenty-six HCW (mean age 41 y, 54% male) with no laboratory evidence of SARS-CoV-2 infection throughout the initial 16-week longitudinal follow up . Neutralising antibody and RBD ELISA data obtained by ROCHE Elecsys anti-SARS-CoV-2 ECLIA following Public Health England (PHE) has been previously published (Manisty et al., 2021a, 2021b; Reynolds et al., 2020, 2021b; Treibel et al., 2020).

### Method details

#### SARS2-CoV-2 immunocomplex assay

##### Bait capture

Ninety six well microtitre plates (Waters Corp) were coated with either Wuhan Hu-1 SARS-CoV-2 spike protein (S1) (Genscript Z03501), Alpha VOC B.1.1.7 SARS-CoV-2 (2019-nCoV) Spike S1 (HV69-70 deletion, N501Y, D614G)-His Recombinant Protein (Sino biological 40591-V08H7), Beta VOC B.1.1351 SARS-CoV-2 Spike protein (S1, E484K, K417N, N501Y, His Tag (Genscript Z03531-1), Delta VOC B.1.617.2 (T19R, G142D, E156G, 157-158 deletion, L452R, T478K, D614G, P681R) Protein (His Tag) (Sino biological 40591-V08H19) or Omicron (B.1.1.529/Omicron) Spike Glycoprotein (S1), Sheep Fc-Tag (HEK293) A67V, H69del, V70del, T95I, G142D, V143del, Y144del, Y145del, N211del, L212I, ins214EPE, G339D, S371L, S373P, S375F, K417N, N440K, G446S, S477N, T478K, E484A, Q493R, G496S, Q498R, N501Y, Y505H, T547K, D614G, H655Y (Native antigen REC32006-100). S1 protein was diluted to 50 μg/mL in PBS and 10 μL added to the bottom of each well. Wells were topped with 140 μL of sodium carbonate/bicarbonate buffer 100 mM at pH 9.6 and then incubated for 12–16 h at 4°C. All further incubations were performed at room temperature (RT) unless stated otherwise. Supernatant was carefully tipped from the wells. Wells were washed with 200 μL of PBS and then incubated for 1 h with 200 μL of blocking solution consisting 1 mg/mL of horse myoglobin (Sigma UK) in PBS followed by 3 washes with PBS. Plate wells were stored with PBS and kept at 4°C until used.

Serum samples were diluted 1:10 in 0.05 mg/mL horse myoglobin (Sigma UK) in PBS and added to S1 protein coated wells for 1 h at 37 °C. Sample was carefully removed and wells were washed once with 200 μL 0.05% Tween 20 in PBS and then 3 times with PBS. For saliva 75 μL of neat saliva was diluted 1:1 in 0.05 mg/mL horse myoglobin in PBS and added to baited wells.

Dried blood spots: 6 mm DBS spots were punched into a 2 mL micro tube and extracted using 175 μL of 0.05 mg/mL Horse myoglobin solution in PBS for 1 h on the shaker. Samples were centrifuged at max rpm on benchtop centrifuge for 10 min. An aliquot 150 μL per reaction was added to the baited well for 1 h.

Dried Saliva spot (lollipop) – 6 mm saliva spots were punched into a 2 mL micro tube and extracted using 250 μL of 0.05 mg/mL Horse myoglobin solution in PBS for 1 h on the shaker. Centrifuge at max rpm on benchtop centrifuge for 10 min. An aliquot of 150 μL per reaction was added to the baited well for 1 h.

#### Immunocomplex protein digestion

Seventy microliters of 0.5% Sodium deoxycholate in 50 mM Ammonium Bicarbonate buffer was added to each well followed by 3 μL of DTT solution (DL-Dithiothreitol (DTT) – 162 mM in 0.5% sodium deoxycholate/50 mM Ammonium bicarbonate buffer). Plates were capped and incubated at 85⁰ C for 15 min with shaking (750 rpm). The plate was left to cool to room temperature before 6 μL of 162 mM Iodoacetamide (IAA) in 0.5% Sodium deoxycholate/50 mM Ammonium bicarbonate buffer was added. Plates were capped and brief shaken and incubated at room temperature for 30 min. Five microliters of trypsin (Sigma) (1 mg/mL in 50 mM Acetic Acid) was added and incubated at 45° C for 30 min. Digestion was halted by addition of 5 μL of 6% TFA and mixed well. Plates were centrifuged for 20 min at 4000 g at 10⁰ C. Fifty microliters are aliquoted into a fresh plate and analysed by LC-MS/MS.

#### Targeted LC-MS/MS analysis

Digested samples were injected onto a Waters 50 mm UPLC Premier ® C18 1.7 μm, 2.1 × 50 mm column operating at 45°C, for chromatographic separation. Mobile phase A consisted of: 0.1% formic acid in water and B: 0.1% formic acid in ACN, pumped at a flow rate of 0.3 mL min-1. The starting conditions of 5% B were kept static for 0.1 min, before initialising the linear gradient to elute and separate peptides over 7.7 min to 40% B. B was linearly increased to 80% over 0.2 min and held for 1 min to wash the column before returning to the initial conditions followed by equilibration for 1 min prior to the subsequent injection. The LC system was coupled to a Waters Xevo-TQ-S triple quadrupole mass spectrometer for multiple reaction monitoring (MRM) detection in positive electrospray ionisation mode. The capillary voltage was set to 2.8 kV, the source temperature to 150°C, the desolvation temperature to 600°C, the cone gas and desolvation gas flows to 150 and 800 L hour-1 respectively. The collision gas consisted of nitrogen and was set to 0.15 mL min-1. The nebuliser operated at 7 bar. The cone energy was set to 35 V and the collision energies varied depending on the optimal settings for each peptide. Optimal peptide transitions for each peptide were selected using Skyline (Figure S5). Transition information of each peptide is given in Table S1.

#### Authentic virus neutralisation assay

SARS-CoV-2 microneutralisation assays were previously reported (Reynolds et al., 2021a, 2021b). VeroE6 cells were seeded in 96-well plates 24 h prior to infection. Duplicate titrations of heat-inactivated participant sera were incubated with 3 × 10^4^ FFU SARS-CoV-2 virus (TCID100) at 37 °C, 1h. Serum/virus preparations were added to cells and incubated for 72h. Surviving cells were fixed in formaldehyde and stained with 0.1% (wt/vol) crystal violet solution (crystal violet was resolubilised in 1% (wt/vol) sodium dodecyl sulphate solution). Absorbance readings were taken at 570 nm using a CLARIOStar Plate Reader (BMG Labtech). Negative controls of pooled pre-pandemic sera (collected prior to 2008), and pooled serum from neutralisation positive SARS-CoV-2 convalescent individuals were spaced across the plates. Absorbance for each well was standardised against technical positive (virus control) and negative (cells only) controls on each plate to determine percentage neutralisation values. IC50s were determined from neutralisation curves. All authentic SARS-CoV-2 propagation and microneutralisation assays were performed in a containment level 3 facility.

### Quantification and statistical analysis

#### Data analysis

Raw LC-MS/MS data was analysed using Skyline open source software (https://skyline.ms/project/home/software/Skyline/begin.view). Peptide identifications were determined from prior analysis of digested serum and immunoglobulin standards (Invitrogen) by a minimum of 6 transitions and matched to *in-silico* spectral library (Prosit) for additional confirmation. Two optimal transitions were used for final MRM analysis. Peptide abundance data were normalised to S1 peptide FASVYAWNR which was present in all variants. For comparison analysis between variant assays ratio values were normalised by Z-Score. Exported data were analysed using Microsoft Excel and Graphpad Prism v9.

Linearity response of IgG up to 250 μg/mL (r > 0.99) was confirmed using a calibration curve using IgG protein standard (Sigma, UK) the maximum observed sample IgG1 value was 157.23 ug/mL well within linear range. LOD and LOQ values (Table S1) were determined by proportion of each IgG subclass of standard. High QC and LQC serum was obtained from vaccinated volunteers. Immunoglobulin CVs were below 30% for all variants apart from low levels for IgG3 and 4. Complement proteins were only detectable in the HQC and showed freeze thaw instability of C4-C9. Therefore complement data for the later emerging Delta variant is not shown.

#### Statistics

For comparison of vaccination groups nonparametric ANOVA (Kruskal Wallis) were used to determine significance. For correlation analyses nonparametric spearman test was used to determine significance and r value.

## Data Availability

• This paper does not report original code. • Any additional information required to re-analyze the data reported in this paper is available from the lead contact upon request.

## Supplemental information

Document S1. Figures S1–S5

Table S1. Targeted LC-MS/MS analysis Immunocomplex peptide transition list. Table of peptide sequences used in the method development and optimization of the multiplex assay. Transition details of masses and optimized cone and collision energies based on a Waters Xevo TQ-S system of each peptide are given related to STAR Methods.

Document S2. Article plus supplemental information

## References

[bib1] Abbasi J. (2021). The flawed science of antibody testing for SARS-CoV-2 immunity. JAMA.

[bib2] Amanat F., Stadlbauer D., Strohmeier S., Nguyen T.H.O., Chromikova V., McMahon M., Jiang K., Asthagiri Arunkumar G., Jurczyszak D., Polanco J. (2020). A serological assay to detect SARS-CoV-2 seroconversion in humans. medRxiv.

[bib3] Anichini G., Terrosi C., Gandolfo C., Gori Savellini G., Fabrizi S., Miceli G.B., Cusi M.G. (2021). SARS-CoV-2 antibody response in persons with past natural infection. N. Engl. J. Med..

[bib4] Dejnirattisai W., Shaw R.H., Supasa P., Liu C., Stuart A.S., Pollard A.J., Liu X., Lambe T., Crook D., Stuart D.I. (2022). Reduced neutralisation of SARS-CoV-2 omicron B.1.1.529 variant by post-immunisation serum. Lancet.

[bib5] Della-Torre E., Lanzillotta M., Strollo M., Ramirez G.A., Dagna L., Tresoldi M., COVID-BioB study group (2021). Serum IgG4 level predicts COVID-19 related mortality. Eur. J. Intern. Med..

[bib6] Dogan M., Kozhaya L., Placek L., Gunter C., Yigit M., Hardy R., Plassmeyer M., Coatney P., Lillard K., Bukhari Z. (2021). SARS-CoV-2 specific antibody and neutralization assays reveal the wide range of the humoral immune response to virus. Commun. Biol..

[bib7] Ebinger J.E., Fert-Bober J., Printsev I., Wu M., Sun N., Prostko J.C., Frias E.C., Stewart J.L., Van Eyk J.E., Braun J.G. (2021). Antibody responses to the BNT162b2 mRNA vaccine in individuals previously infected with SARS-CoV-2. Nat. Med..

[bib8] Ferrante A., Beard L.J., Feldman R.G. (1990). IgG subclass distribution of antibodies to bacterial and viral antigens. Pediatr. Infect. Dis. J..

[bib9] Gaebler C., Wang Z., Lorenzi J.C.C., Muecksch F., Finkin S., Tokuyama M., Cho A., Jankovic M., Schaefer-Babajew D., Oliveira T.Y. (2021). Evolution of antibody immunity to SARS-CoV-2. Nature.

[bib10] Jarlhelt I., Nielsen S.K., Jahn C.X.H., Hansen C.B., Pérez-Alós L., Rosbjerg A., Bayarri-Olmos R., Skjoedt M.O., Garred P. (2021). SARS-CoV-2 antibodies mediate complement and cellular driven inflammation. Front. Immunol..

[bib11] Jayasekera J.P., Moseman E.A., Carroll M.C. (2007). Natural antibody and complement mediate neutralization of influenza virus in the absence of prior immunity. J. Virol..

[bib12] Ju B., Zhang Q., Ge J., Wang R., Sun J., Ge X., Yu J., Shan S., Zhou B., Song S. (2020). Human neutralizing antibodies elicited by SARS-CoV-2 infection. Nature.

[bib13] Kim P., Gordon S.M., Sheehan M.M., Rothberg M.B. (2021). Duration of SARS-CoV-2 natural immunity and protection against the delta variant: a retrospective cohort study. Clin. Infect. Dis..

[bib14] Kunnakkadan U., Nag J., Kumar N.A., Mukesh R.K., Suma S.M., Johnson J.B. (2019). Complement-mediated neutralization of a potent neurotropic human pathogen, chandipura virus, is dependent on C1q. J. Virol..

[bib15] Manisty C., Otter A.D., Treibel T.A., McKnight Á., Altmann D.M., Brooks T., Noursadeghi M., Boyton R.J., Semper A., Moon J.C. (2021). Antibody response to first BNT162b2 dose in previously SARS-CoV-2-infected individuals. Lancet.

[bib16] Manisty C., Treibel T.A., Jensen M., Semper A., Joy G., Gupta R.K., Cutino-Moguel T., Andiapen M., Jones J., Taylor S. (2021). Time series analysis and mechanistic modelling of heterogeneity and sero-reversion in antibody responses to mild SARSCoV-2 infection. EBioMedicine.

[bib17] Mehlhop E., Nelson S., Jost C.A., Gorlatov S., Johnson S., Fremont D.H., Diamond M.S., Pierson T.C. (2009). Complement protein C1q reduces the stoichiometric threshold for antibody-mediated neutralization of West Nile virus. Cell Host Microbe.

[bib18] van der Neut Kolfschoten M., Schuurman J., Losen M., Bleeker W.K., Martínez-Martínez P., Vermeulen E., den Bleker T.H., Wiegman L., Vink T., Aarden L.A. (2007). Anti-inflammatory activity of human IgG4 antibodies by dynamic Fab arm exchange. Science.

[bib19] Nie J., Li Q., Wu J., Zhao C., Hao H., Liu H., Zhang L., Nie L., Qin H., Wang M. (2020). Quantification of SARS-CoV-2 neutralizing antibody by a pseudotyped virus-based assay. Nat. Protoc..

[bib20] Pastorino B., Touret F., Gilles M., de Lamballerie X., Charrel R.N. (2020). Heat inactivation of different types of SARS-CoV-2 samples: what protocols for biosafety, molecular detection and serological diagnostics?. Viruses.

[bib21] Patil H.P., Rane P.S., Shrivastava S., Palkar S., Lalwani S., Mishra A.C., Arankalle V.A. (2021). Antibody (IgA, IgG, and IgG subtype) responses to SARS-CoV-2 in severe and nonsevere COVID-19 patients. Viral Immunol..

[bib22] Perez-Toledo M., Faustini S.E., Jossi S.E., Shields A.M., Marcial-Juarez E., Kanthimathinathan H.K., Allen J.D., Watanabe Y., Goodall M., Willcox B.E. (2021). SARS-CoV-2-specific IgG1/IgG3 but not IgM in children with pediatric inflammatory multi-system syndrome. Pediatr. Allergy Immunol..

[bib23] Public Health England (2021). https://www.gov.uk/government/publications/covid-19-laboratory-evaluations-of-serological-assays.

[bib24] Reynolds C.J., Pade C., Gibbons J.M., Otter A.D., Lin K.M., Muñoz Sandoval D., Pieper F.P., Butler D.K., Liu S., Joy G., COVIDsortium Investigators (2022). Immune boosting by B.1.1.529 **(**omicron) depends on previous SARS-CoV-2 exposure. Science.

[bib25] Reynolds C.J., Gibbons J.M., Pade C., Lin K.M., Sandoval D.M., Pieper F., Butler D.K., Liu S., Otter A.D., Joy G. (2021). Heterologous infection and vaccination shapes immunity against SARS-CoV-2 variants. Science.

[bib26] Reynolds C.J., Pade C., Gibbons J.M., Butler D.K., Otter A.D., Menacho K., Fontana M., Smit A., Sackville-West J.E., Cutino-Moguel T. (2021). Prior SARS-CoV-2 infection rescues B and T cell responses to variants after first vaccine dose. Science.

[bib27] Reynolds C.J., Swadling L., Gibbons J.M., Pade C., Jensen M.P., Diniz M.O., Schmidt N.M., Butler D.K., Amin O.E., Bailey S.N.L. (2020). Discordant neutralizing antibody and T cell responses in asymptomatic and mild SARS-CoV-2 infection. Sci. Immunol..

[bib28] Saxena S.K., Kumar S., Ansari S., Paweska J.T., Maurya V.K., Tripathi A.K., Abdel-Moneim A.S. (2022). Characterization of the novel SARS-CoV-2 Omicron (B.1.1.529) variant of concern and its global perspective. J. Med. Virol..

[bib29] Schiela B., Bernklau S., Malekshahi Z., Deutschmann D., Koske I., Banki Z., Thielens N.M., Würzner R., Speth C., Weiss G. (2018). Active human complement reduces the zika virus load via formation of the membrane-attack complex. Front. Immunol..

[bib30] Smit N.P.M., Ruhaak L.R., Romijn F.P.H.T.M., Pieterse M.M., van der Burgt Y.E.M., Cobbaert C.M. (2021). The time has come for quantitative protein mass spectrometry tests that target unmet clinical needs. J. Am. Soc. Mass Spectrom..

[bib31] Treibel T.A., Manisty C., Burton M., McKnight Á., Lambourne J., Augusto J.B., Couto-Parada X., Cutino-Moguel T., Noursadeghi M., Moon J.C. (2020). COVID-19: PCR screening of asymptomatic health-care workers at London hospital. Lancet.

[bib32] Vasantha S., Coelingh K.L., Murphy B.R., Dourmashkin R.R., Hammer C.H., Frank M.M., Fries L.F. (1988). Interactions of a nonneutralizing IgM antibody and complement in parainfluenza virus neutralization. Virology.

[bib33] Wang L., Cheng G. (2022). Sequence analysis of the emerging SARS-CoV-2 variant Omicron in South Africa. J. Med. Virol..

[bib34] Yang T.J., Yu P.Y., Chang Y.C., Liang K.H., Tso H.C., Ho M.R., Chen W.Y., Lin H.T., Wu H.C., Hsu S.T.D. (2021). Effect of SARS-CoV-2 B.1.1.7 mutations on spike protein structure and function. Nat. Struct. Mol. Biol..

[bib35] Yu H.Q., Sun B.Q., Fang Z.F., Zhao J.C., Liu X.Y., Li Y.M., Sun X.Z., Liang H.F., Zhong B., Huang Z.F. (2020). Distinct features of SARS-CoV-2-specific IgA response in COVID-19 patients. Eur. Respir. J..

[bib36] Yu X., Wei D., Xu W., Li Y., Li X., Zhang X., Qu J., Yang Z., Chen E. (2022). Reduced sensitivity of SARS-CoV-2 Omicron variant to antibody neutralization elicited by booster vaccination. Cell Discov..

[bib37] Zhou D., Dejnirattisai W., Supasa P., Liu C., Mentzer A.J., Ginn H.M., Zhao Y., Duyvesteyn H.M.E., Tuekprakhon A., Nutalai R. (2021). Evidence of escape of SARS-CoV-2 variant B.1.351 from natural and vaccine-induced sera. Cell.

