# Supplementary material for: Quantitative, multiplexed, targeted proteomics for ascertaining variant specific SARS-CoV-2 antibody response
Source: Cell Rep Methods. 2022 Aug 12;2(9):100279. doi: 10.1016/j.crmeth.2022.100279 (PMC9372021; doi:10.1016/j.crmeth.2022.100279)
Supplement: Document S1. Figures S1–S5 [file mmc1.pdf]

Cell Reports Methods, Volume *2*

Supplemental information

Quantitative, multiplexed, targeted proteomics for ascertaining variant specific SARS-CoV-2 antibody response

Ivan Doykov, Tomas Baldwin, Justyna Spiewak, Kimberly C. Gilmour, Joseph M. Gibbons, Corinna Pade, Catherine J. Reynolds, Áine McKnight, Mahdad Noursadeghi, Mala K. Maini, Charlotte Manisty, Thomas Treibel, Gabriella Captur, Marianna Fontana, Rosemary J. Boyton, Daniel M. Altmann, Tim Brooks, Amanda Semper, UK COVIDsortium Investigators, James C. Moon, Kevin Mills, and Wendy E. Heywood

Supplementary figures

Supplementary figure S1. Multivariate analysis of the immunocomplex response to SARS-CoV2 infection or vaccine.

Supplementary figure S2. Proof of principle of application of assay to other tissues.

Supplementary figure S3. Spearman correlation matrix of components of the immunocomplex and corresponding neutralising antibodies .

Supplementary Figure S4. C1q relationship to IgG1 and neutralising antibodies and Response of triple vaccinated HCW to the Omicron BA.1 VoC.

Supplementary figure S5. Representative overlaid chromatograms of multiple transitions for each peptide used in the multiplex assay

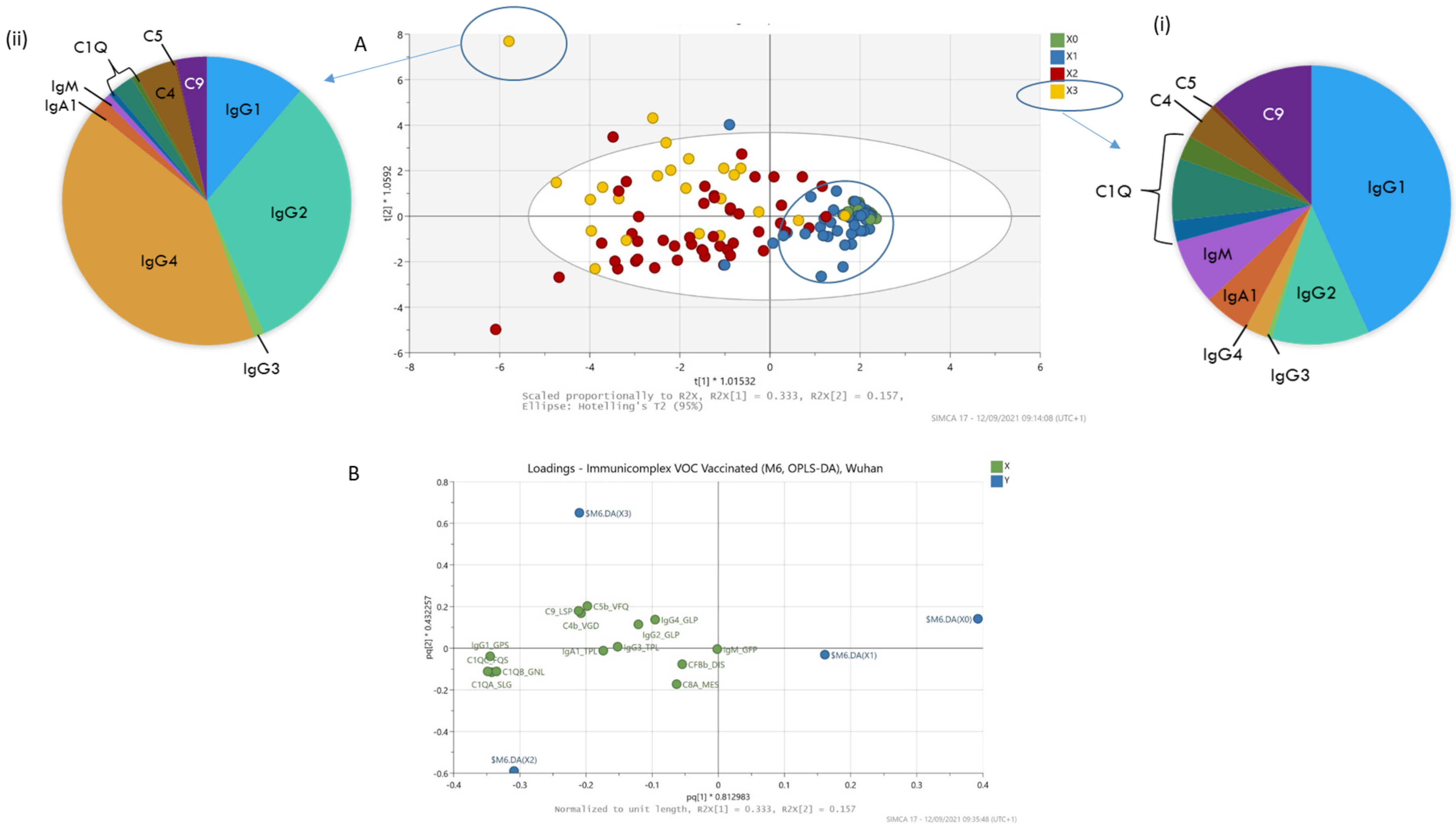

Figure S1. **Multivariate analysis of the immunocomplex response to SARS-CoV-2 infection or vaccine related to figure 1.**

Figure S1 A shows a multivariate OPLS-DA plot of all vaccine groups coloured according to their exposure to SARS CoV-2 vaccination and/or infection. The X0 (no exposure) and X1 (single exposure by infection or vaccination) groups (circled cluster), both cluster away from the doubly vaccinated or single vaccine + infection (X2) group and vaccinated plus previous SARS-CoV-2 infection groups (X3 exposure). X2 and X3 have a greater degree of variation indicating individuals have a large varied response to exposure. Pie chart (i) shows a typical profile based on mean values for the double vaccinated + pre-infection group with dominating IgG1. Pie chart (ii) shows a profile of an outlier with a profile dominated by IgG4 and IgG2 demonstrating individual heterogeneity in the immunocomplex. Figure S2B shows the loading plot of the variables that drive the distribution of the groups in Figure S2A. These results indicate that IgG1 and C1Q are the variables that have the greatest influence and importance on separating the X2 and X3 each group from X0 and X1.

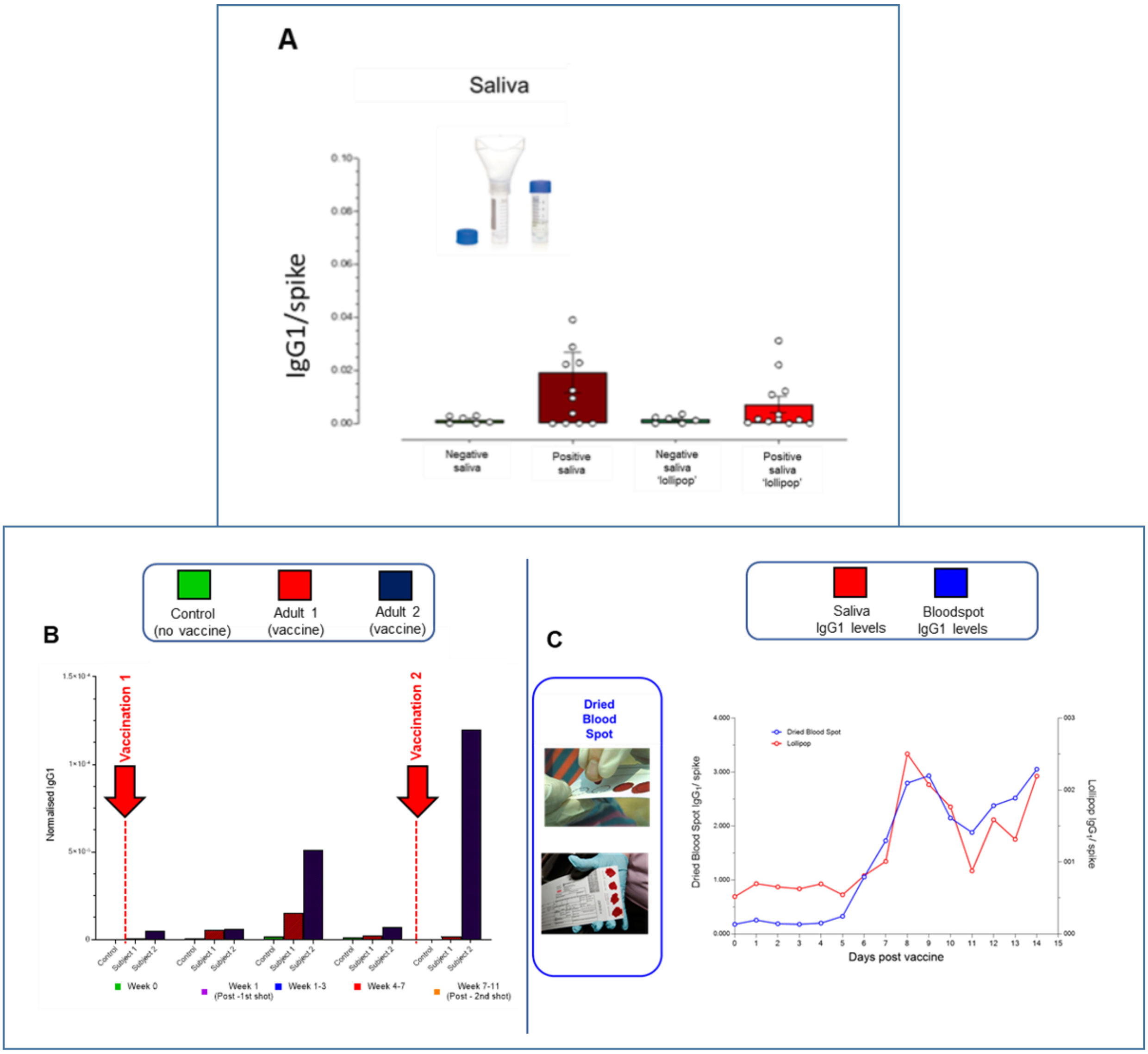

Supplementary Figure S2. **Proof of principle of application of assay to other tissues related to STAR methods**

Figure S2A demonstrates how the assay can be used to measure IgG1 levels in both saliva and saliva 'lollipops' 40-60 days after patients have tested positive for SARS COV2. These IgG1 levels are similar to those observed in individuals after receiving one vaccination or exposure (50% of patients have increased antibody levels). These results indicate that simple collection of saliva via absorbent filter paper used in the collection of bloodspots gives comparable results to neat saliva whilst simplifying the collection and storage of samples.

Figure S2B demonstrates how the assay has be used to measure IgG1 levels in the saliva of patients undergoing a full vaccination protocol. Saliva IgG1 levels were monitored in 3 patients over a 12-week period. As expected no increase in IgG1 levels were observed in the control patient who did not receive a vaccine but both patient 1 and 2 both demonstrated an increases in IgG1 after vaccination. Interestingly, patient 1 although demonstrated an increase in IgG1 levels after the first vaccination, no increases were observed after the second vaccination. In contrast, Patient 2 demonstrated a significant increase in IgG1 levels after both vaccinations. C shows the IgG1 levels in paired bloodspots and saliva measured daily, from the same patient for two weeks post vaccination. Although antibody levels are significantly lower in saliva than blood, relative antibody levels closely follow each other, indicating bloodspots and saliva 'lollipops' maybe be useful for high throughput, home testing of patients.

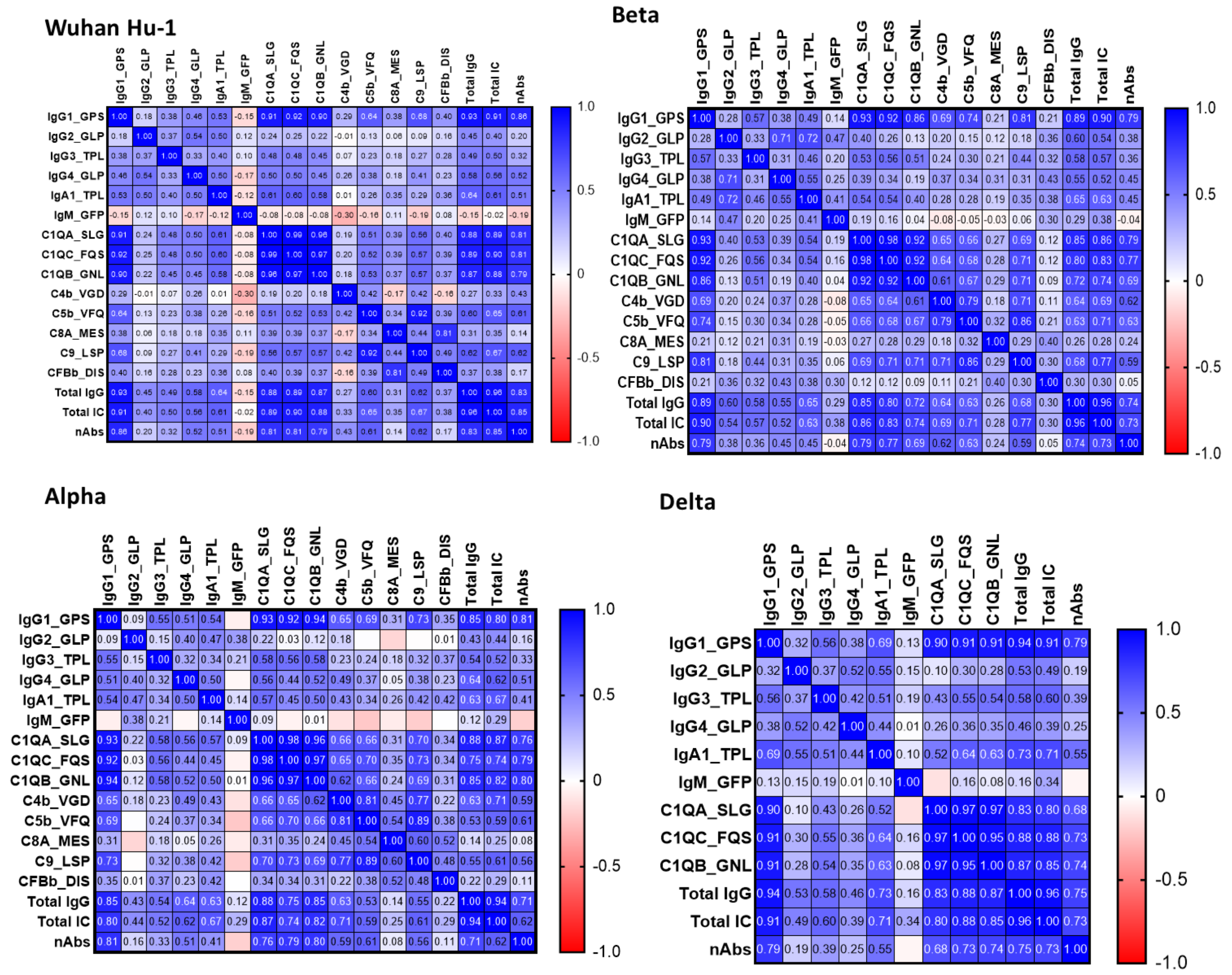

Figure S3. Spearman correlation matrix of components of the immunocomplex and corresponding neutralising antibodies related to figure 2. Total Ig is the sum of IgG 1-4 and total IC is sum of all the components of the immunocomplex assay.

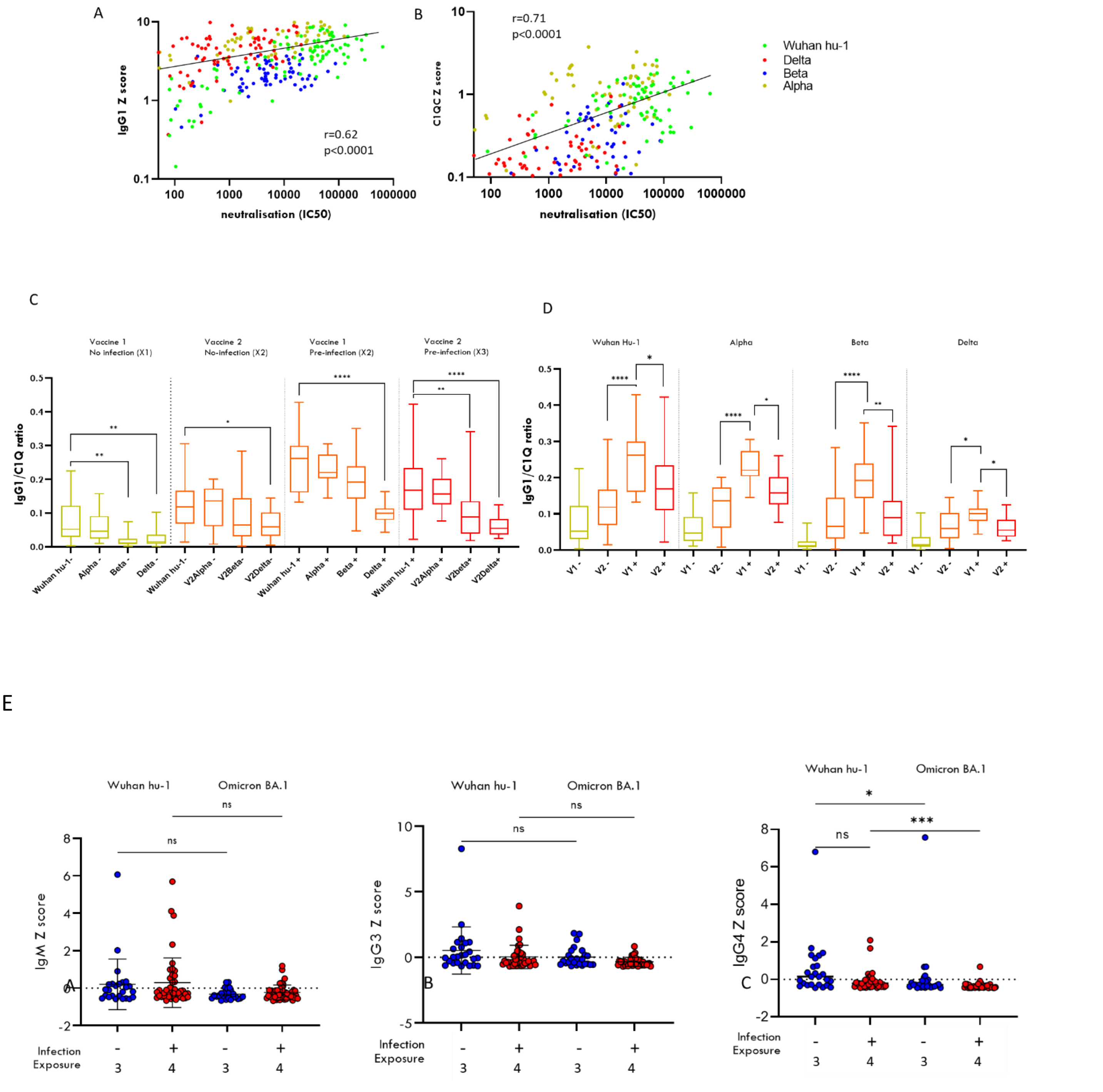

Supplementary Figure S4. **C1Q relationship to IgG1 and neutralising antibodies related to figure 4 and response of triple vaccinated HCW to the Omicron BA.1 VoC related to figure 5 (A)** Shows correlation of IgG1 to nAbs for all vaccination groups and for all variants together (**B)** Shows stronger correlation of C1Q to nAbs for all vaccination groups and for all variants together. The ratio of IgG1 to C1Q (**C)** shows an increased ratio in responseto previous infection at first vaccination which is less at second vaccination. (**D**) shows the ratio across variants with significant reduction for Beta and Delta. Groups coloured according to exposure yellow first exposure, orange second exposure and red third exposure. Boxplots (ii) show min- max with mean +/- 1SD. Significance determined by non-parametric ANOVA. **(E)** Comparison of triple vaccinated with or without prior infection against S1 protein from Wuhan hu-1 or Omicron BA.1 infection naïve n= 25 (blue) prior infected n=36 (red). Significance determined by non parametric Kruskal Wallis test.

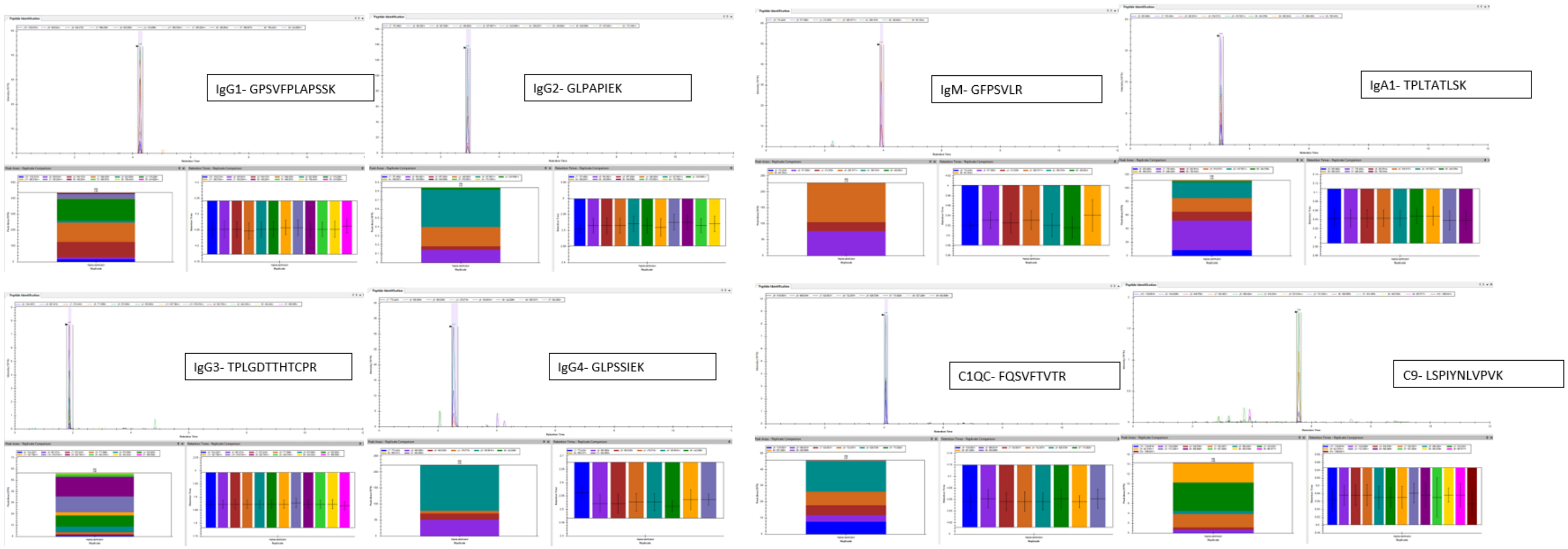

Figure S5 related to STAR methods. **Representative overlaid chromatograms of multiple transitions for each peptide used in the multiplex assay**. The abundance and retention times are also indicated in below panels. Two best transitions are typically selected based on highest abundance and lack of co-eluting interfering peaks observed in sample matrix.
