# Supplementary material for: Quantitative, multiplexed, targeted proteomics for ascertaining variant specific SARS-CoV-2 antibody response
Source: Cell Rep Methods. 2022 Aug 12;2(9):100279. doi: 10.1016/j.crmeth.2022.100279 (PMC9372021; doi:10.1016/j.crmeth.2022.100279)
Supplement: Table S1. Targeted LC-MS/MS analysis — Immunocomplex peptide transition list. Table of peptide sequences used in the method development and optimization of the multiplex assay. Transition details of masses and optimized cone and collision energies based on a Waters Xevo TQ-S system of each peptide are given related to STAR Methods. [file mmc2.pdf]

Protein	Sequence	precursor.mz	product.m_z	cone_voltage	collision_energy	ion_name
C4b	VGDTLNLNLR	557.81	1015.55	35	20	y9
C4b	VGDTLNLNLR	557.81	629.37	35	20	y5
C4BPA	YTCLPGYVR	564.78	864.44	35	20	y7
C4BPA	YTCLPGYVR	564.78	591.32	35	20	y5
C5b	VFQFLEK	455.76	811.43	35	16	y6
C5b	VFQFLEK	455.76	664.37	35	16	y5
C8A	MESLGITSR	497.26	733.42	35	17	y7
C8A	MESLGITSR	497.26	533.30	35	17	y5
C8G	SLPVSDSVLSGFEQR	540.95	723.34	35	18	y6
C8G	SLPVSDSVLSGFEQR	540.95	636.31	35	18	y5
C9	LSPIYNLVPVK	621.88	832.49	35	22	y7
C9	LSPIYNLVPVK	621.88	521.82	35	22	y9
CFBb	DISEVVTPR	508.27	787.43	35	18	y7
CFBb	DISEVVTPR	508.27	571.36	35	18	y5
PON1	IFFYDSENPPASEVLR	628.64	771.44	35	21	y7
PON1	IFFYDSENPPASEVLR	628.64	434.75	35	21	y8
C3	AEDLVGK	366.20	660.36	35	12	y6
C3	AEDLVGK	366.20	531.31	35	12	y5
VTN	FEDGVLDPDYPR	711.83	875.43	35	25	y7
VTN	FEDGVLDPDYPR	711.83	647.31	35	25	y5
Total IgG	NQVSLTCLVK	581.32	919.53	35	20	y8
Total IgG	NQVSLTCLVK	581.32	820.46	35	20	y7
IgG1	GPSVFPLAPSSK	593.83	699.40	35	21	y7
IgG1	GPSVFPLAPSSK	593.83	418.23	35	21	y4
IgG2	GLPAPIEK	412.75	486.29	35	14	y4
IgG2	GLPAPIEK	412.75	327.69	35	14	y6
IgG3	TPLGDTTHTCPR	452.55	579.27	35	15	y10
IgG3	TPLGDTTHTCPR	452.55	522.73	35	15	y9
IgG4	GLPSSIEK	415.73	563.30	35	14	y5
IgG4	GLPSSIEK	415.73	330.68	35	14	y6
IgA1	TPLTATLSK	466.28	620.36	35	16	y6
IgA1	TPLTATLSK	466.28	415.75	35	16	y8
IgM	GFPSVLR	388.23	474.30	35	13	y4
IgM	GFPSVLR	388.23	286.18	35	13	y5
Kappa	VDNALQSGNSQESVTEQDSK	712.66	893.42	35	24	y8
Kappa	VDNALQSGNSQESVTEQDSK	712.66	707.32	35	24	y6
Lambda	YAASSYLSLTPEQWK	872.43	988.51	35	31	y8
Lambda	YAASSYLSLTPEQWK	872.43	687.35	35	31	y5
SARS-CoV2 spike	GVYYPDK	421.21	685.32	35	15	y5
SARS-CoV2 spike	GVYYPDK	421.21	359.19	35	15	y3
SARS-CoV2 spike	FASVYAWNR	557.28	808.41	35	20	y6
SARS-CoV2 spike	FASVYAWNR	557.28	709.34	35	20	y5
SARS-CoV2 spike	QIAPGQTGK	450.25	658.35	35	16	y7
SARS-CoV2 spike	QIAPGQTGK	450.25	587.31	35	16	y6
SARS-CoV2 spike	VGGNYNYLYR	609.80	1119.52	35	21	y9
SARS-CoV2 spike	VGGNYNYLYR	609.80	728.37	35	21	y5
C1QA	SLGFCDTTNK	571.76	942.40	35	20	y8
C1QA	SLGFCDTTNK	571.76	738.31	35	20	y6
C1QC	FQSVFTVTR	542.79	809.45	35	19	y7
C1QC	FQSVFTVTR	542.79	809.42	35	19	b7
C1QB	GNLCVNLMR	538.77	792.39	35	19	y6
C1QB	GNLCVNLMR	538.77	632.35	35	19	y5
